# Membrane prewetting by condensates promotes tight-junction belt formation

**DOI:** 10.1038/s41586-024-07726-0

**Published:** 2024-08-07

**Authors:** Karina Pombo-García, Omar Adame-Arana, Cecilie Martin-Lemaitre, Frank Jülicher, Alf Honigmann

**Affiliations:** 1 Max Planck Institute of Molecular Cell Biology and Genetics, Dresden, Germany; 2 Max Planck Institute for the Physics of Complex Systems, Dresden, Germany; 3 Center for Systems Biology Dresden, Dresden, Germany; 4 Cluster of Excellence Physics of Life, TU Dresden, Dresden, Germany; 5 Technische Universität Dresden, Biotechnologisches Zentrum, Center for Molecular and Cellular Bioengineering, Dresden, Germany; 6 Present Address: Rosalind Franklin Institute, Oxford, United Kingdom

**Keywords:** Membrane structure and assembly, Cell adhesion

## Abstract

Biomolecular condensates enable cell compartmentalization by acting as membraneless organelles^1^. How cells control the interactions of condensates with other cellular structures such as membranes to drive morphological transitions remains poorly understood. We discovered that formation of a tight-junction belt, which is essential for sealing epithelial tissues, is driven by a wetting phenomenon that promotes the growth of a condensed ZO-1 layer^2^ around the apical membrane interface. Using temporal proximity proteomics in combination with imaging and thermodynamic theory, we found that the polarity protein PATJ mediates a transition of ZO-1 into a condensed surface layer that elongates around the apical interface. In line with the experimental observations, our theory of condensate growth shows that the speed of elongation depends on the binding affinity of ZO-1 to the apical interface and is constant. Here, using PATJ mutations, we show that ZO-1 interface binding is necessary and sufficient for tight-junction belt formation. Our results demonstrate how cells exploit the collective biophysical properties of protein condensates at membrane interfaces to shape mesoscale structures.

## Abstract (web summary)

Experimental and theoretical results demonstrate that tight-junction formation depends on the growth of a condensed layer of ZO-1 proteins at the interface of the apical and lateral membrane, providing insight into self-assembly of complex mesoscale structures in cells.

## Main

Tight junctions are supramolecular adhesion complexes that control the paracellular flux of solutes by forming diffusion barriers between cells^3–5^. Junctional assembly is initiated by condensation of cytosolic scaffold ZO proteins at cell–cell contact sites that over time elongate and fuse around the apical cell perimeters into a continuous belt that seals the tissue^2,6^. How the nucleated junctional membrane condensates undergo changes in composition and are shaped into a continuous tight-junction belt is unclear. Physical wetting phenomena of biomolecular condensates on cellular structures such as membranes can, in principle, drive morphological changes through forces emerging at the interfaces of condensates^7^. Depending on the strength of binding of phase-separating components to a membrane, transitions between a dilute adsorbed and condensed surface layer can occur below the saturation concentration for bulk phase separation. Such transitions are called prewetting transitions^8–11^. How cells tune the properties of condensates^12,13^ to control the interactions with biological surfaces^14,15^ is poorly understood.

Here we uncover a striking example of how tight-junction condensates elongate along the apical membrane interface. We show that cells tune the molecular interactions of junctional condensates with the apical polarity protein PATJ to shape and position the junctional belt at the apical membrane interface. After condensate nucleation, the formation of a continuous tight-junction belt is driven by the growth of ZO-1 surface condensates along the apical membrane interface through a prewetting transition. Here, condensates elongate with a constant velocity, which follows from the theory of growth of a dense phase into a dilute phase^16^. This wetting phenomenon depends on the molecular interactions of cytoplasmic scaffold proteins with the polarized cell membrane and collectively guides the mesoscale shape of the tight-junction complex in space and time.

## Time-resolved junction proximity proteomics

**Extended Data Fig. 1:**
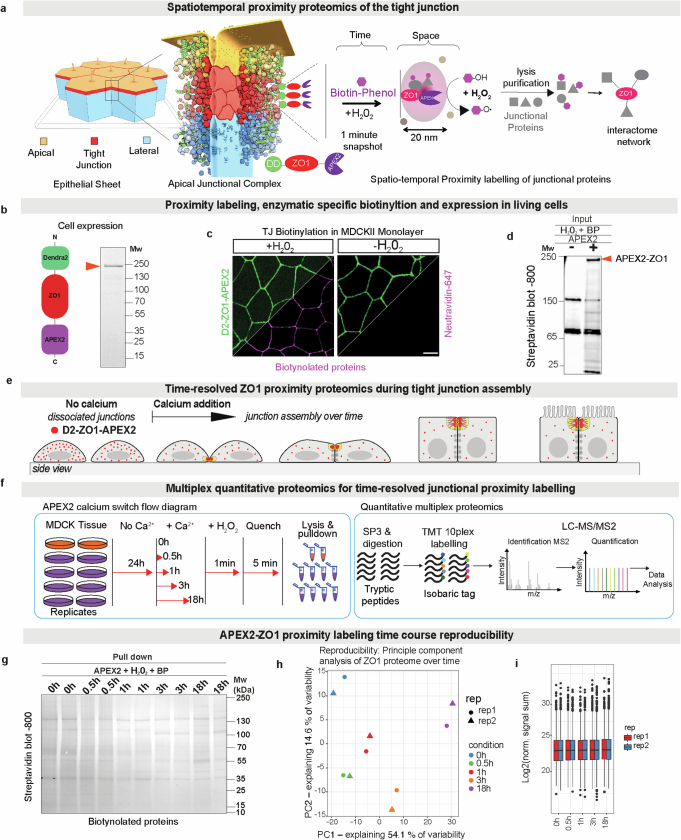
Experimental pipeline of time-resolved ZO-1-APEX2 proximity proteomics. (a) Scheme of labelling D2-ZO-1-APEX2 to identify protein interactions at the apical junctional complex. After activation for 1 min, proteins in proximity ( < 20 nm) to the ZO-1 get biotinylated and are identified using mass spectrometry. (b) D2-ZO-1-APEX2 genetic construct and protein gel of the fusion protein in MDCK-II cells with a Mw ~ 250 kDa. (c) Localization of the D2-ZO-1-APEX2 at the tight junction with H_2_O_2_ specific activation and biotinylation of APEX2. Scale bar 10 µm. (d) Activation of APEX2 and biotinylation pattern (+/− H_2_O_2_) by western blot. (e) Scheme of tight junction assembly and D2-ZO1-APEX2 localization during the calcium switch in epithelia. (f) Time-resolved proximity labelling, biotinylated proteins purified by streptavidin pull down, SP3 enrichment, digestion, TMT labelling and quantitative mass spectrometry. (g) Western blot of the biotinylated proteins after pull-down at different time points used for proteomics. (h) Principal component analysis (PCA) plots of the proteome cross the different time points of the ZO-1-APEX2 proximity proteomics. The coloured dots and triangle represent the n = 2 biological replicate and the different colour the time points (red, blue, orange, violet and green). PCA show the clusters changed in a reproducible manner. (i) Protein intensities of all samples at different time points show that protein content did not change. Plot shows the normalized signal sum in the biological replicates across all time points. Data represents center lines medians, box borders represent the interquartile range (IQR), and whiskers extend to ±1.5× the IQR; outliers are shown as black dots. Panels b,d show representative images of n = 3 and g of n = 2 biological replicates. Source Data

To understand how the tight-junction belt is assembled and positioned, we combined APEX2 proximity proteomics^17^ of the main junctional scaffold protein ZO-1 (Extended Data Fig. 1a–d) with a calcium switch tissue-formation assay^18,19^. This combination allowed us to synchronize the initiation of junction assembly in the entire tissue by addition of calcium to the culture medium and quantify the time evolution of the junctional proteome during the assembly process using proximity proteomics.

**Fig. 1:**
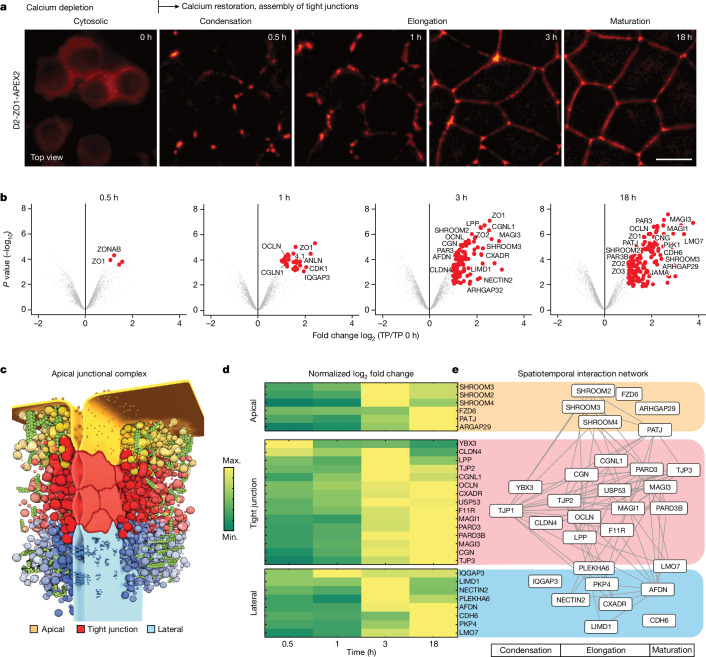
Proximity proteomics of ZO-1 condensates during tight-junction formation. **a**, Live imaging of tight-junction belt formation after calcium switch of MDCK-II monolayer expressing Dendra2–ZO-1–APEX2 (*n* = 3 biological replicates). The morphological stages of the ZO-1 belt are indicated. **b**, Volcano plots displaying log_2_ fold change in abundance of biotinylated proteins proximal to ZO-1 compared with time point zero against −log_10_ *P* values at different stages of tight-junction formation. *P* values were calculated using moderated *t*-statistics after Benjamini–Hochberg adjustment using the limma package in R for each time point. Proteins with FDR < 0.05 and fold change ≥ 2 were considered to be significantly enriched and are depicted in red. Background proteins are shown in grey. Selected junctional proteins are annotated (see Extended Data Fig. 1 for setup and controls for APEX2). **c**, Scheme of the apical–junction complex showing three subcompartments: apical (yellow), tight junction (red) and lateral (blue) along the epithelial membrane. **d**, Heat map of normalized log_2_ fold change values for selected apical, tight-junction and lateral proteins over time (Supplementary Table 2). **e**, Spatiotemporal protein interaction network relating the known positions of proteins (apical, junctional, lateral) to the arrival kinetics revealed by the ZO-1–APEX2 proteomics. Scale bar, 10 µm.

On the basis of the dynamics of ZO-1 distribution, we subdivided the junction assembly process into four morphological stages (Fig. 1a). Between 0 h and 0.5 h, cytoplasmic ZO-1 condensed at nascent cell–cell contacts. Between 1 h and 3 h, ZO-1 nucleated membrane condensates elongated and fused into a continuous belt. Finally, between 3 h and 18 h, the junctional belt and cell shapes equilibrated into a stable confluent epithelial monolayer. To capture the molecular changes that accompany the morphological transitions, we performed ZO-1 proteomics proximity-labelling and mass spectrometry analysis at 0 h, 0.5 h, 1 h, 3 h and 18 h after calcium switch (Extended Data Fig. 1e–i and Supplementary Note 2).

To analyse the changes in the ZO-1 interactome as a function of tight-junction assembly time, we calculated the fold change in protein abundance with respect to the calcium-depleted state (0 h) (Fig. 1b and Supplementary Tables 1 and 3). The number of proteins proximal to ZO-1 significantly increased during the assembly process, showing that composition after nucleation of the condensates is strongly remodelled over time. Taken together, the results of our ZO-1–APEX2 proteomics proximity profiling provide a quantitative map of protein arrival kinetics during tight-junction assembly. We use this dataset as a resource to understand the molecular underpinning of tight-junction belt formation.

## ZO-1 proximity to apical proteins

The tight junction is known to be compartmentalized into different functional regions, which have been suggested to be important for assembly and positioning (Fig. 1c). Interactions with adherens junctions in the lateral membrane have been implicated in initiation of junction assembly at cell–cell contacts and in providing mechanical robustness^20–22^. Interactions of the tight junction with the apical membrane are thought to be important for positioning and signalling of the junctional belt^23,24^.

To understand when the interactions with the lateral and apical compartments are established, we grouped our time-resolved proximity proteomics data according to their known compartment identity and analysed their recruitment kinetics^25^ (Fig. 1c–e and Supplementary Table 2). This analysis showed that the first adhesion receptors that are enriched around ZO-1 are occludin and claudin-4, and later nectin-2 and JAM-A; these are classified as core tight-junction proteins^26–28^. Interactions with cadherin complexes (CDH6) are established at a later stage, possibly via afadin (3–18 h) (Supplementary Note 3). Most junctional scaffold proteins such as MAGI-3, PAR3 and CGN are recruited into junctional condensates between 1 and 3 h (refs. ^23,29^). Notably, components of the apical compartment such as members of the SHROOM family, which are cytoskeletal adaptors involved in cell shape regulation, and the polarity protein PATJ are recruited during the junction spreading phase (approximately 3 h)^30,31^.

Taken together, the results of the temporal proximity analysis show that ZO-1 condensation starts around a core set of tight-junction proteins. Subsequently, ZO-1 condensates contact apical components, around the time they elongate around the cell perimeter, suggesting a functional connection between these morphological and molecular processes.

## Tight-junction recruitment dynamics

**Extended Data Fig. 2:**
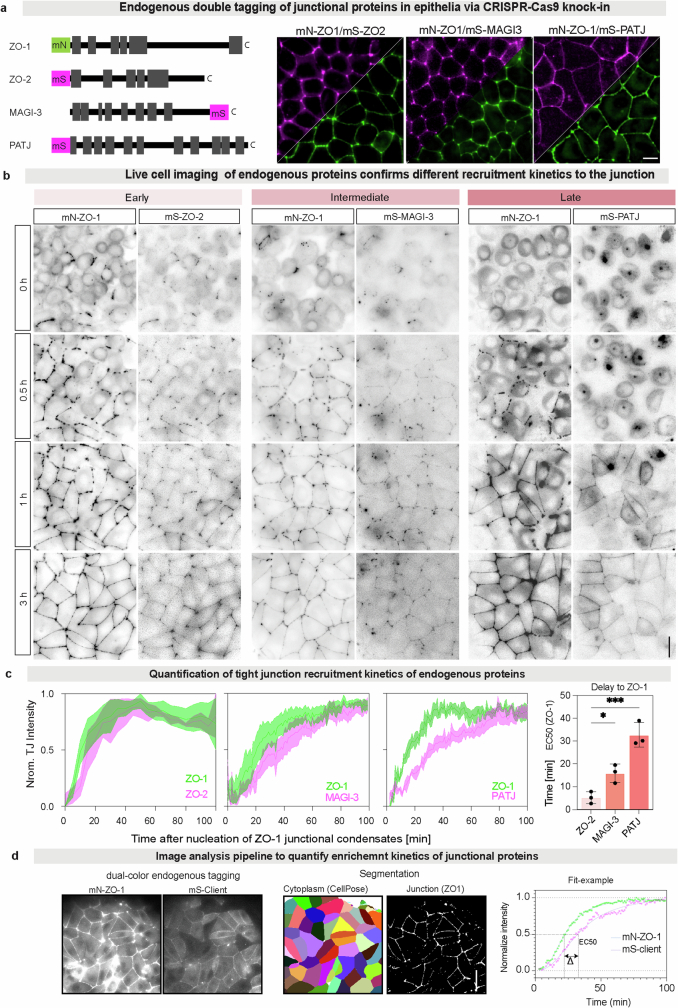
Live-imaging of endogenous proteins show recruitment kinetics to ZO-1 condensates. (a) Double fluorescence tagging via CRISPR/Cas9 mediated knock-in of mN-ZO1 (green) and mS-ZO2, mS-MAGI3 or mS-PATJ (magenta) in MDCK-II cells. Dual-color imaging of the cell lines in confluent monolayers confirmed mN-ZO-1 co-localization with tagged proteins. (b) Calcium switch imaging of the double tagged cell lines. Snapshots of same time steps as used for the APEX2 proximity proteomics are shown with inverted color maps (0.5 h,1 h,3 h). Scale bar 10 µm. (c) Quantification of protein enrichment in mN-ZO-1 condensates compared to the cytoplasm over time. Plots show the normalized enrichment of mS-ZO-2, mS-MAGI-3 and mS-PATJ (magenta) at the tight junction compared to mN-ZO-1 (green), data shows mean ± SD of n = 3 independent experiments. Kinetics were fitted with a Hill binding model. The half-time plot of recruitment compared to mN-ZO-1. Significant comparison in reference to ZO-2 (early) using a one-way ordinary ANOVA test correct for multiple comparations with Dunnett (not significant (n.s), p  >  0.12; *p  <  0.034, **p  <  0.002. (d) Image analysis pipeline showing time-resolved two-color imaging of junction assembly. Cells were segmented using CellPose. ZO-1-condensates were segmented using local intensity threshold of mN-ZO1. Junction enrichment was calculated locally for every cell as the ratio of junctional and cytoplasmic fluorescence intensity. Quantification of junction arrival time by fitting junction enrichment kinetics with Hill binding model. Delta between ZO-1 and each client protein represents the different arrival time. Panels a, b show representative images of n = 3 biological replicates. Source Data

To directly visualize and verify individual protein arrival kinetics during tight-junction assembly, we used quantitative live imaging of endogenously tagged proteins identified in the proteomics analysis (Fig. 1d). We tagged one candidate from the early stage (ZO-2), one from the intermediate stage (MAGI-3) and one from the late stage (PATJ) (Extended Data Fig. 2a and Supplementary Note 4).

Live imaging of the two-colour cell lines using the calcium switch assay allowed us to quantify the kinetics of protein arrival to mN-ZO-1 condensates during tight-junction assembly (Extended Data Fig. 2b–d). The kinetics of the junction enrichment ratio showed that mS-ZO-2 became rapidly enriched after ZO-1 condensation, with a half time (*t*_1/2_) of approximately 5 min. By comparison, mS-MAGI-3 was recruited more slowly and reached saturation later, with a *t*_1/2_ of around 18 min. Finally, mS-PATJ recruitment was delayed even longer and showed visible enrichment only around the time of junction spreading, with a *t*_1/2_ of approximately 35 min (Extended Data Fig. 2c).

Taken together, the results of live imaging confirmed the protein recruitment kinetics established by time-resolved APEX2 proteomics. Importantly, we confirmed that the polarity protein PATJ arrives at the junction at around the time of junctional condensate elongation, indicating that interactions of ZO-1 condensates with the apical membrane may be important for the transition into a continuous junctional belt.

## PATJ arrival correlates with elongation

**Fig. 2:**
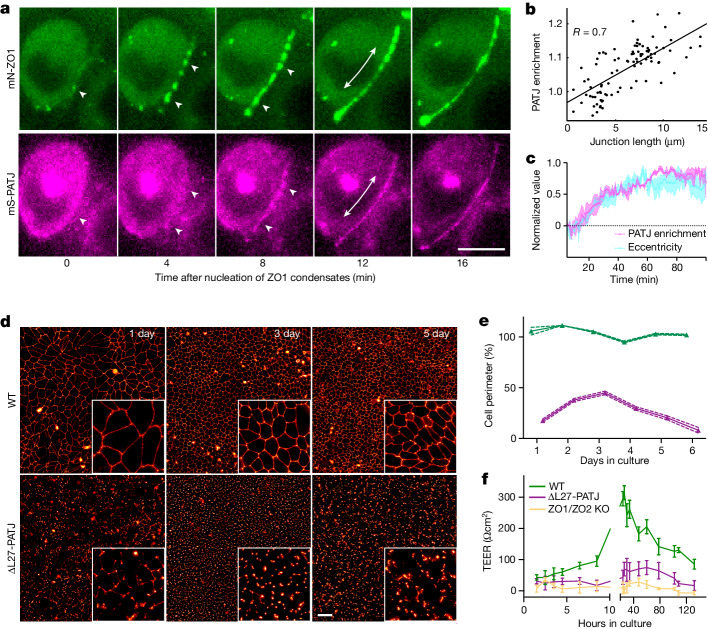
PATJ recruitment to junctional condensates is correlated with elongation. **a**, Dual-colour live imaging of endogenous mN-ZO-1 and mS-PATJ at cell–cell contacts during tight-junction formation. Triangles indicate nucleation of membrane condensates and arrows indicate condensate elongation (Supplementary Video 3 and Supplementary Note 4). Representative images of *n* = 4 biological replicates). **b**, Quantification of PATJ enrichment in ZO-1 condensates versus elongation of condensates, showing a strong correlation (*R* = 0.7, 101 condensates, *n* = 4 independent experiments). **c**, Quantification of normalized PATJ enrichment and normalized condensate eccentricity over time show that both processes follow similar kinetics. Data are the mean ± s.e.m. of *n* = 3 biological replicates. **d**, Live imaging of endogenous mN-ZO-1 in WT and ∆L27-PATJ MDCK-II monolayers over the course of 1, 3 and 5 days after seeding of *n* = 3 biological experiments for each time point. **e**, Quantification of ZO-1 belt coverage of the cell perimeter (%) in growing WT and ∆L27-PATJ MDCK-II monolayers over the course of 6 days after seeding. WT cells formed a continuous ZO-1 belt from day 1. In ∆L27- PATJ tissue, ZO-1 remained fragmented even after 6 days of culture. Data are shown as the mean and s.d. of *n* = 3 biological replicates (Extended Data Fig. 3b). **f**, Transepithelial electrical resistance measurements of WT, ∆L27-PATJ and ZO-1/2-knockout MDCK-II monolayers in transwell dishes measured for 1 h after seeding until 6 days (mean ± s.d. of *n* = 3 biological replicates). Scale bars, 5 µm (**a**), 10 µm (**d**). TEER, transepithelial resistance. Source Data

To test the hypothesis that PATJ mediates the elongation of junctional condensates, we analysed how the enrichment of PATJ in the condensates relates to their extension (Fig. 2a and Supplementary Video 2). The analysis showed a strong positive correlation (*R* = 0.7) between the extension of ZO-1 condensates and PATJ enrichment; that is, PATJ became significantly enriched as the condensate elongated (Fig. 2b). In addition, plotting the time evolution of the average eccentricity of junctional condenses together with the junctional enrichment of PATJ clearly showed that both properties follow the same kinetics: round ZO-1 condensates deformed into an eccentric elongated shape at the same time that PATJ concentration increased (Fig. 2c). The correlation between elongation of ZO-1 condensates and PATJ enrichment and the similar timing support the idea that PATJ mediates the extension of the condensates.

## Condensate elongation requires PATJ

**Extended Data Fig. 3:**
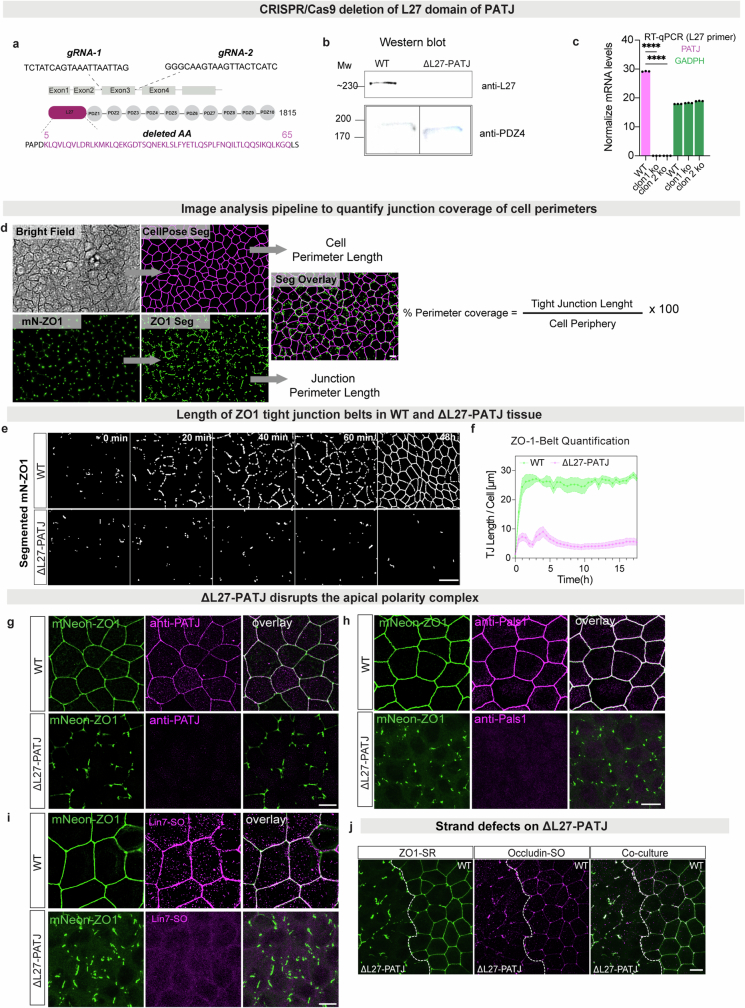
PATJ is required for tight junction formation. (a) Deletion of L27 domain of PATJ via CRISPR/Cas9 in the background of mN-ZO-1 knock in. Guides used to remove exon 3 and sequencing confirming the deletion of the first exon and frame shift leading to an early stop codon in two independent clones. (b) Western Blot showing PATJ protein levels in WT and ∆L27-PATJ cells using anti-L27 or anti-PDZ4 domain antibodies. Antibody against the N-terminal L27 domain of PATJ, encoded by the first exon, confirmed deletion of this domain (up). Antibody against the C-terminal PDZ4 show a remaining truncated protein with a smaller size of ~ 40 kDa (low), (see Supporting Fig. 1). (c) qPCR showing the mRNA levels of PATJ in WT and two independents cell clones of ∆L27-PATJ deleted L27 domain encoded by the first exon. Data shows mean ± SD, statistical analysis was done using an unpair t test (not significant (n.s), p < 0.00001****). (d) Segmentation pipeline to quantify the coverage of cell perimeters by mN-ZO-1 condensates using CellPose for the cell perimeter on phase contrast images and local intensity thresholding and skeletonization in Fiji for mN-ZO1. (e) Segmented and skeletonized mN-ZO1 during calcium switch quantifying tight junction per cell in WT vs ∆L27-PATJ MDCK-II during 48 h. (f) Quantification of tight junction length per cell for segmented panel e, data shows mean ± SEM of n = 7 monolayers n > 50 cells. (g) Staining of PATJ (magenta) and ZO-1(green) in WT vs ∆L27-PATJ MDCK-II. Staining of Pals1 (magenta) and ZO-1(green) in WT vs ∆L27-PATJ MDCK-II. (i) Staining of Lin7 (magenta) and ZO-1(green) in WT vs ∆L27-PATJ MDCK-II. (j) Staining of Occudin (magenta) and ZO-1(green) in WT vs ∆L27-PATJ MDCK-II co-culture cells. Panels show representative images of n = 2 (b), n = 10 (d) and n = 3 biological replicates (e, f, g,h,I,j). Scale bar is 20 µm (d, e) and 10 µm for (g,h,I,j). Source Data

Previous studies have shown that PATJ is a multidomain scaffold protein that binds to the apical polarity Crumbs complex through its amino-terminal L27 (ref. ^32^). PATJ depletion by RNA interference has been shown to cause a delay in tight-junction formation^24,33^. To investigate the role of PATJ in elongation of ZO-1 condensates and tight-junction belt formation, we deleted the N-terminal L27 domain using CRISPR–Cas9 in the background of the mN-ZO-1 knock-in MDCK-II (Extended Data Fig. 3a–c,g and Supplementary Table 4).

Imaging of mN-ZO-1 distribution in confluent ∆L27-PATJ monolayers revealed disruption of the tight-junction belt compared with wild-type (WT) tissue (Fig. 2d); that is, the belt was often disconnected and ZO-1 appeared to be enriched at tricellular contacts (Supplementary Video 1). In line with this observation, quantification of the average belt length per cell and the normalized cell perimeter coverage of ZO-1 in WT and ∆L27-PATJ tissues over the course of 10 days showed overall strong reduction of belt length and perimeter coverage in ∆L27-PATJ cells (Fig. 2e and Extended Data Fig. 3d–f). In addition, ∆L27-PATJ tissue had significantly reduced transepithelial electrical resistance compared with WT (Fig. 2f). The loss of PATJ also led to loss of other apical polarity complex proteins (PALS-1 and LIN-7) from the tight junctions, as well as fragmentation of the tight-junction protein occludin (Extended Data Fig. 3h–j) but E-cadherin distribution was not affected (Extended Data Fig. 7f). Taken together, these results indicate that PATJ is required for the elongation of ZO-1 condensates along the apical interface and the formation of a functional tight-junction barrier.

## PATJ mediates apical connection of ZO-1

**Fig. 3:**
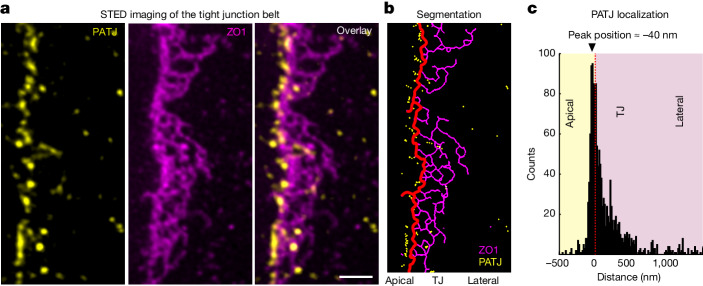
PATJ localizes to the apical interface of the ZO-1 belt. **a**, Dual-colour super-resolution STED images of PATJ (yellow) and ZO-1 (magenta) at cell–cell interface of MDCK-II cysts. ZO-1 forms a network structure reminiscent of tight-junction strands. PATJ is enriched as clusters around the apical interface of the ZO-1 network. Representative images of *n* = 12 independent cell–cell interfaces. See Extended Data Fig. 4 for imaging set-up and cell–cell interface orientation. **b**, Segmentation of the ZO-1 network and PATJ clusters. The most apical ZO-1 strand is shown in red. The distance of PATJ clusters from the apical ZO-1 strand was quantified (Extended Data Fig. 4a,b). **c**, Histogram of the distances of PATJ from the apical ZO-1 strand. PATJ localization showed strong enrichment just apical of ZO-1. (Distribution contains 979 localizations of *n* = 12 independent cell–cell interfaces. The mode of the distribution is 40 nm apical of the ZO-1 strand.) Scale bar, 500 nm. Source Data

**Extended Data Fig. 4:**
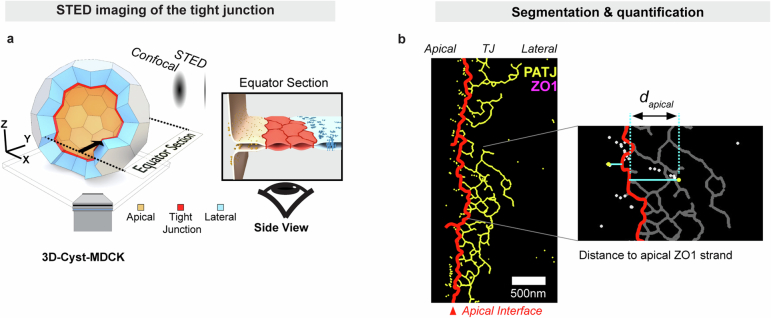
STED super-resolution imaging of the apical interface. (a) Combining 3D tissue culture of MDCK-II cysts with STED microscopy enables super- resolution imaging of cell-cell interfaces. At the equator cell-cell interfaces are oriented parallel to the high-resolution axis (XY) of the microscope. (b) 2-color STED imaging of PATJ (yellow) and ZO-1 (magenta) in MDCK-II cysts reveal that ZO-1 forms a network structure reminiscent of tight junction strands. PATJ is enriched as clusters at the apical interface of the ZO-1 network.

To better understand how PATJ promotes the growth of junctional condensates along the apical perimeter, we performed two-colour stimulated emission depletion (STED) super-resolution microscopy to determine the ultrastructure of PATJ and ZO-1 at the tight junction. To this end, we used a combination of two-dimensional STED microscopy with three-dimensional tissue culture, which enabled imaging of cell–cell interfaces in the high-resolution plane of the microscope^34^ (Extended Data Fig. 4a,b). STED imaging of fully formed junctions showed that ZO-1 formed a condensed belt at the apical cell–cell interface (Fig. 3a). Towards the lateral side, the belt ZO-1 formed a network-like structure reminiscent of tight-junction strands observed by freeze-fracture electron microscopy^35^. Notably, PATJ was strongly enriched at the apical interface of the condensed ZO-1 junctional network, in line with previous observations^25,36^. We found that PATJ formed clusters around the most apical strand of the ZO-1 network. Quantification of the nearest-neighbour distance between the most apical ZO-1 strand and PATJ clusters resulted in a distribution with a peak 40 nm apical of the ZO-1 belt and an exponential-like decay towards the lateral membrane (Fig. 3b,c). Thus, PATJ is in close proximity to ZO-1, but it is mostly excluded from the core of the ZO-1 condensate in mature tissue.

Taken together, the results of the super-resolution analysis show that PATJ forms an interface between the apical membrane and the ZO-1 network, supporting the idea that PATJ mediates interactions of ZO-1 condensates with the apical membrane interface. The distance analysis between PATJ and ZO-1 showed that PATJ is in very close proximity to ZO-1 but remains locally excluded from the core of the condensate. This observation suggests two types of interaction underlying complex formation between ZO-1 and PATJ: an attractive interaction that mediates colocalization; and a repulsive interaction that drives segregation. On the basis of previous work, we speculated that the attractive interaction could be facilitated by short-range binding of membrane-bound PATJ to ZO proteins^24,25,37–39^. Repulsion could be the consequence of PATJ being part of the apical polarity complex, which would prevent mixing with the lateral membrane^40,41^. The combination of long-range segregation between lateral and apical domains with short-range binding interactions could provide a mechanism that leads to accumulation of ZO-1 along the apical–lateral interface.

## Condensates grow via a prewetting transition

**Fig. 4:**
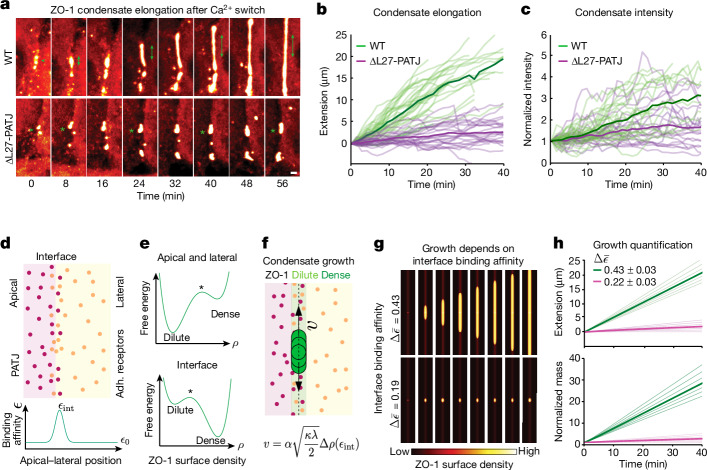
Thermodynamics of a prewetting transition explain observed elongation dynamics. **a**, ZO-1 condensates nucleate at the cell–cell interface (arrows), elongate and fuse into a continuous belt (double-sided arrow) in WT cells. In ∆L27-PATJ cells, condensates nucleate but fail to elongate along the interface (stars). **b**, Traces showing extensions of single condensate in WT and ∆L27-PATJ (Extended Data Fig. 5a–c). Bold lines indicate the mean of *n* = 32 WT and n = 24 ∆L27-PATJ traces. **c**, Total condensate intensity normalized to initial intensity of each condensate in WT and ∆L27-PATJ. Bold lines indicate the mean of *n* = 31 WT and n = 19 ∆L27-PATJ traces. **d**, Sketch of the polarized membrane domains: apical, lateral and interface. Bottom diagram depicts the binding affinity of ZO-1 to the membrane, which is highest at the interface. **e**, Thermodynamic free energy as a function of ZO-1 surface density. For the apical and lateral membrane (top), the dilute phase has the lowest free energy, whereas at the apical–lateral interface (bottom), owing to a higher binding affinity, the condensed phase has the lowest free energy. **f**, Sketch of elongation of a nucleated ZO-1 condensate along the apical–lateral membrane interface via a prewetting transition. The derived growth velocity $v$ of surface condensates as a function of binding affinity ${{\epsilon }}_{\mathrm{int}}$ to the apical interface is shown (Extended Data Fig. 5e,l and Supplementary Note 1). **g**, Numerical solutions to the surface condensation theory for two different values of the relative binding affinity $\Delta \bar{{\epsilon }}$ of ZO-1 to the interface. Dynamics for $\Delta \bar{{\epsilon }}=0.43\,$ (top) and $\Delta \bar{{\epsilon }}=0.19$ (bottom). **h**, Theoretical condensate extension (top) and mass increase (bottom) as functions of binding affinity. Solid green and magenta lines correspond to relative binding affinities $\Delta \bar{{\epsilon }}=0.43$ and $\Delta \bar{{\epsilon }}=0.22$, respectively. The dim lines correspond to relative binding affinities $\Delta \bar{{\epsilon }}=\mathrm{0.46,\; 0.45,\; 0.44,\; 0.42,\; 0.41,\; 0.4}$ and $\Delta \bar{{\epsilon }}=\mathrm{0.25,\; 0.24,\; 0.23,\; 0.21,\; 0.20,\; 0.19}$, respectively. **a**, **b** and **c** show *n* = 4 biological replicates. Scale bar, 1 µm. Source Data

**Extended Data Fig. 5:**
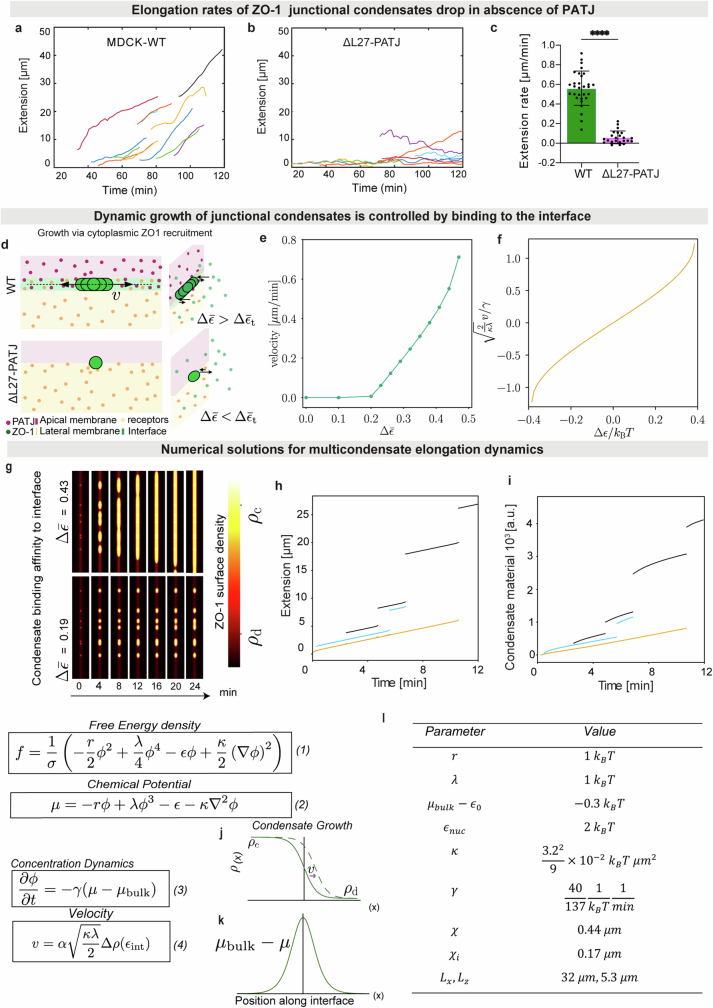
Prewetting velocity is determined by ZO-1 binding affinity to the apical interface. (a) Single mN-ZO-1 condensate extension dynamics after calcium switch of WT. (b) Single mN-ZO-1 condensate extension dynamics after calcium switch of ∆L27-PATJ. (c) Quantification of the elongation velocity (extension rate) of individual condensates from WT (r = 0.5 ± 0.2 µm/min) and ∆L27-PATJ (r = 0.05 ± 0.1 µm/min) after calcium switch from Fig. 4a (mean ± SD). (d) Scheme of condensate nucleation and extension in WT and ∆L27-PATJ. WT condensates nucleate and elongate along the interface with velocity *v* (left) recruiting ZO-1 molecules from the bulk (right) when relative binding affinity, $\Delta \bar{{\epsilon }}$ is larger than the threshold value, $\Delta {\bar{{\epsilon }}}_{t}$, for which the droplets do not extend. Nucleated ∆L27-PATJ condensates do not elongate, because relative binding affinity to the apical interface is too low $\Delta \bar{{\epsilon }} < \Delta {\bar{{\epsilon }}}_{t}$. (e) Numerical calculation of the velocity of single condensates as a function of the relative ZO-1 binding affinity to the interface region. The velocity decreases as the binding affinity decreases until it reaches zero. (f) Analytical curve for the extension velocity in a one-dimensional system as function of the binding affinity to the apical interface relative to the bulk chemical potential. (g) Snapshots of numerical solutions of concentration dynamics equation. Numerical solutions for relative binding affinity $\Delta \bar{{\epsilon }}=0.43$ (top), observing linear extension over time and $\Delta \bar{{\epsilon }}=0.19$ (bottom) with no extension. (h) Extension of multiple condensates over time with $\Delta \bar{{\epsilon }}=0.43$ where each extends linearly fusing into a belt. (i) Total amount of material over time of multiple condensates for $\Delta \bar{{\epsilon }}=0.43$ show a linear correlation. Total intensity of each condensate shown as solid-colored line and fusing shows an intensity jump reducing the curves number. (j) Table containing the parameters and values used for the numerical calculations. Panels (a, b) are representative of n = 12 condensates (c) of n = 30 condensates of n = 4 biological replicates. Source Data

**Extended Data Fig. 6:**
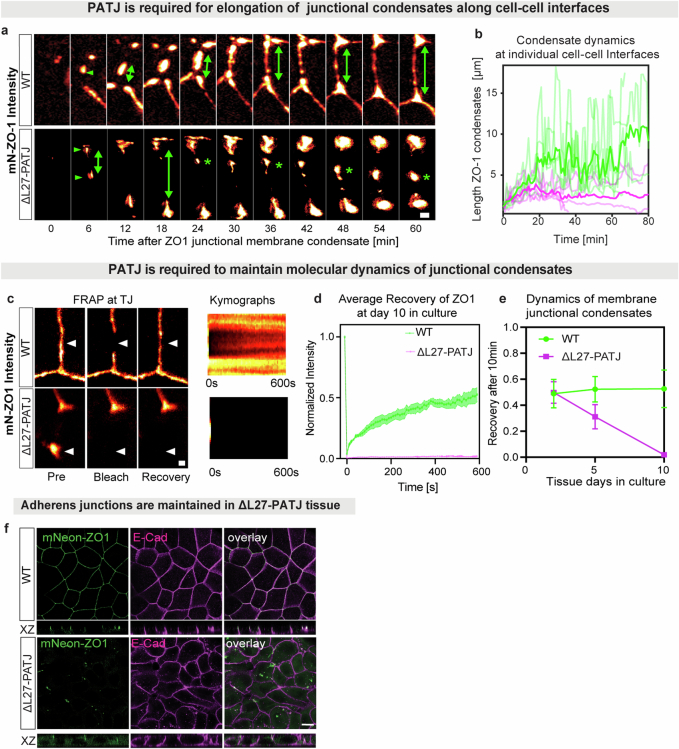
Dynamics of ZO-1 condensates during tight junction formation. (a) ZO-1 junctional condensate dynamics at single cell-cell interfaces (green arrows) after calcium switch, elongate and fuse into a continuous belt in WT (double side arrow) (Supplementary Video 3) but fail to elongate and fuse in ∆L27-PATJ (green stars), Scale bar 1 µm. (b) Quantification of the length of ZO-1 condensates over time. Time 0 min refers to the nucleation of ZO-1 condensates on the membrane. In WT cells the length of ZO-1 condensates on average increases over time to a length of 10 µm (bold green). Single condensates show strong fluctuations of length due to transient fusion and fission events along the cell perimeter (light green). In ∆L27-PATJ cells the average length of ZO-1 condensates remains below 5 µm (bold pink) and the fluctuations due to elongation and fission are strongly reduced (light pink). (c) FRAP experiment showing pre and post bleach images and kymographs over 600 s at 10 days. mN-ZO-1 was bleached at the membrane, and recovery was measured at room temperature over time. Kymographs show that ZO-1 recovered rapidly from the cytoplasm in WT. Reduced recovery was observed in ∆L27-PATJ cells indicating that dynamics of ZO-1 in condensates are slower representative of n = 4 biological replicates. (d) Quantification of recovery dynamics of mN-ZO-1 in junctional condensates of FRAP experiments. Averaged recovery curves shown of n = 10 independent measurement (mean ± SD). Scale bar 1 µm. (e) Quantification of mobile fraction after 10 min recovery over the course of day 2, 5 and 10. Plot shows mean ± SD of n = 4–9 cell junctions from n = 3 biological replicates. (f) Staining of E-Cadherin in WT and ∆L27-PATJ confluent monolayers expressing mN-ZO1, representative of n = 3 biological replicates. Scale bar 10 µm. Panels (a,b) show n = 5 cells from n = 3 biological replicates. Source Data

To understand how PATJ enables elongation of ZO-1 condensates along the apical interface, we quantified the dynamics of ZO-1 condensates in WT and ∆L27-PATJ tissues after calcium switch (Extended Data Fig. 6a and Supplementary Video 3). Quantification of the elongation of single junctional condensates (Fig. 4a) showed that in WT cells, condensate elongation on average increased over time up to 10–20 µm per cell after 40 min (Fig. 4b). During the elongation, we often observed fusion and fission of condensates, causing fluctuations in condensate length (Extended Data Fig. 6b). When focusing on the extension events without fusion and fission, we found that the extension, *E*, followed a linear scaling, *E*(*t*) ∝ *t* (Fig. 4b, Extended Data Fig. 5a and Supplementary Video 4). In addition, we quantified the amount of ZO-1 over time per condensate by measuring the sum of the intensity per condensate, *I* (Fig. 4c). Similar to the extension, we found that the amount of ZO-1 increased linearly over time, *I*(*t*) ∝ *t*, indicating that further ZO-1 molecules were sequestered to the condensate during the extension process (Fig. 4c). Comparing the elongation process in WT and ∆L27-PATJ tissues (Fig. 4a–c and Extended Data Fig. 5b), we found that nucleation of condensates at nascent cell–cell adhesion sites was hardly affected by ∆L27-PATJ (Extended Data Fig. 5a,b and Supplementary Video 3). However, the loss of PATJ from the membrane inhibited the elongation of nascent condensates along the apical interface. The average extension rate of condensates was 0.53 ± 0.14 µm min^−1^ in WT tissue, whereas it slowed significantly to 0.02 ± 0.07 µm min^−1^ in ∆L27-PATJ tissue (Extended Data Fig. 5c). The amount of ZO-1 also increased significantly more slowly compared with WT tissue, albeit the difference was less pronounced compared with the extension data.

To understand the physical mechanism of elongation, we compared the experimental results with the thermodynamics of surface condensate growth^10,42^. We used the observed condensate elongation dynamics to discriminate between a simple binding scenario, wetting and prewetting. We found that a prewetting scenario was the most appropriate physical description (Supplementary Note 1). We considered the following experimental observations. ZO-1 concentration in the bulk (cytoplasm) is below the saturation concentration for phase separation^2,43^; that is, the system is in a prewetting regime where nucleation and growth of condensation does not happen in the bulk cytoplasm but can occur on the membrane surface owing to preferential interactions^10^. The membrane is polarized into an apical domain containing PATJ molecules and a lateral domain containing adhesion receptors, which both have low binding affinity *ϵ* to ZO-1 (Fig. 4d). The binding affinity of ZO-1 to the membrane, *ϵ*, depends on the position on the membrane and is highest at the apical–lateral interface (Fig. 4d, bottom panel). In our model, we described a transition between a dilute layer and a condensed layer using the free energy density, *f*, as a function of the dimensionless composition variable *ϕ*, which is related to the surface density *ρ* of ZO-1 (equation 1 in Extended Data Fig. 5 and Supplementary Note 1). The free energy exhibits a double-well shape, in which the wells represent dilute and dense surface states of ZO-1 (Fig. 4e). In the apical and lateral domains, the ZO-1 binding affinity is low, ${\epsilon }=\,{{\epsilon }}_{0}$; in this case, the dilute phase has the lowest free energy. At the apical–lateral interface, ZO-1 binding affinity is higher, ${\epsilon }=\,{{\epsilon }}_{\mathrm{int}}$, and the condensed phase has the lowest free energy (Fig. 4e, Supplementary Note 1 (for a detailed description of the model) and Extended Data Fig. 5).

Initially, this model exhibited dilute binding of ZO-1 on the membrane, which is slightly enriched along the apical interface (Fig. 4f). To overcome the free energy barrier of surface condensate formation, a nucleation event is required. In the model, we introduced nucleation sites that were defined as small circular regions at the apical interface with higher binding affinity, ${\epsilon }=\,{{\epsilon }}_{{\rm{nuc}}}+\,{{\epsilon }}_{0}$, that allowed surface condensates to overcome the energy barrier in the double-well free energy (Fig. 4e and Supplementary Note 1). After nucleation, owing to an imbalance of the chemical potential of ZO-1 in the bulk (cytoplasm), *μ*_bulk_, and at the condensate edges, *μ*, the condensate extended along the apical interface by recruiting ZO-1 molecules from the bulk to its edge (Fig. 4f, equations 2 and 3 in Extended Data Fig. 5, and Extended Data Fig. 5j,k). The elongation process resulted in a constant growth velocity $v$, which depended on the ZO-1 binding affinity at the interface, ${{\epsilon }}_{\mathrm{int}}$, relative to the ZO-1 bulk chemical potential *μ*_bulk_ via $\Delta {\epsilon }={{\epsilon }}_{{\rm{int}}}+{\mu }_{{\rm{bulk}}}$; note that *μ*_bulk_ < 0. For increasing ${{\epsilon }}_{\mathrm{int}}$, the growth velocity $v$ increased (Extended Data Fig. 5e,f and equation 4 in Extended Data Fig. 5). After a nucleation event, two-dimensional condensates did not grow if ${{\epsilon }}_{\mathrm{int}}$ was below a critical value (Extended Data Fig. 5d,e).

Numerical solutions of the concentration dynamics of this model in two dimensions (equation 3 in Extended Data Fig. 5) reproduced key experimental results for extending ZO-1 condensates. After nucleation, condensates extended with a constant velocity along the apical interface when the binding affinity ${{\epsilon }}_{\mathrm{int}}$ was above a threshold value (top row in Fig. 4g and Extended Data Fig. 5d,e). In this case, the condensate extension and mass grew linearly with time (Fig. 4h). We could relate the binding affinity of ZO-1 to the amount of PATJ at the apical interface in the experimental system. Below a critical binding affinity, the extension velocity of a newly nucleated condensate was zero, and the condensate failed to elongate as experimentally observed in ΔPATJ tissue (Fig. 4g,h, Extended Data Fig. 5d,e and Supplementary Video 4). This remained true in the case of multiple nucleated condensates (Extended Data Fig. 5g–i and Supplementary Video 5).

Taken together, these results show that a prewetting transition at the apical interface is in line with the experimentally observed elongation dynamics of nucleated ZO-1 condensates. The dependence of the extension velocity on the strength of binding of ZO-1 to the apical interface suggests a mechanistic link between the role of PATJ as an apical connector for ZO-1 and its ability to mediate growth of condensates along the apical interface.

## Apical binding rescues belt formation

**Fig. 5:**
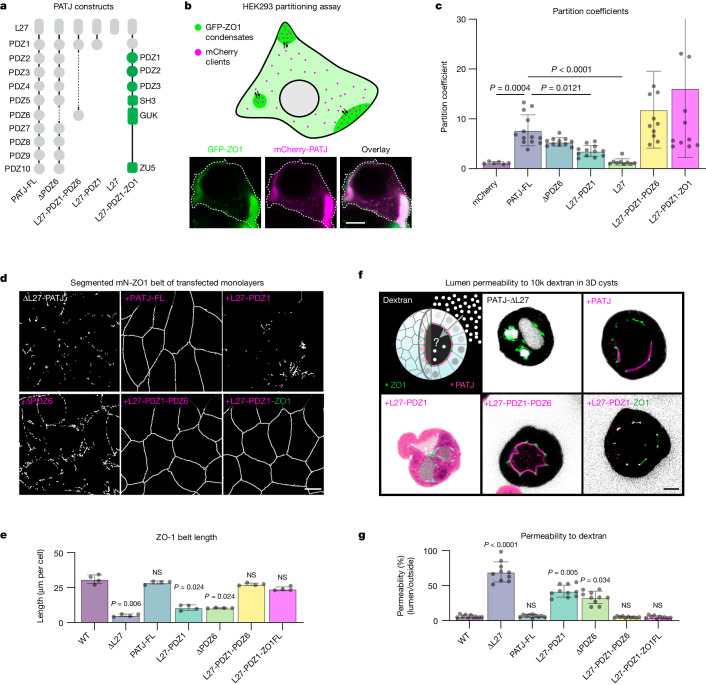
Interactions of ZO-1 condensates with the apical interface are required for tight-junction formation. **a**, Domain structure of PATJ constructs used for partitioning and rescue experiments. **b**, Scheme of the HEK293 partitioning assay to quantify interaction of the PATJ mutants with ZO-1 condensates. Representative example of the partitioning of PATJ on ZO-1 condensates of *n* = 3 biological replicates. **c**, Partitioning of PATJ constructs labelled with mCherry into GFP–ZO-1 condensates in HEK293 cells. Data are mean ± s.d. of *n* = 11–14 condensates of *n* = 3 independent experiments. **d**, Segmented maximum projections of mN-ZO-1 junctional belts of ∆L27-PATJ monolayers transfected with the PATJ truncation constructs, representative of *n* = 3 biological replicates. **e**, Quantification of segmented tight-junction length per cell (*n* > 50 cells) for the different PATJ constructs (mean ± s.d. from *n* = 3 biological replicates). **f**, Transepithelial permeability of ∆L27-PATJ MDCK-II cysts transfected with PATJ constructs. Three-dimensional cysts grown in Matrigel were incubated with 10k dextran-Alexa647. Confocal middle sections of cysts are shown, representative of *n* = 3 biological replicates. **g**, Quantification of transepithelial permeability of 10k dextran from the outside to the lumen of three-dimensional WT and ∆L27-PATJ MDCK-II cysts expressing rescue constructs. Green indicates mN-ZO-1 and magenta the PATJ rescue construct. Data are shown as the mean ± s.d. of *n* = 10 cysts from *n* = 3 biological replicates. Statistical analyses used Kruskal–Wallis test with post hoc Dunn’s multiple-comparison test. Scale bars, 5 µm (**b**), 10 µm (**d**,**f**). Source Data

Our data so far indicated that ZO-1 and PATJ interact at the apical interface, which promotes condensate elongation into a functional tight-junction belt via a wetting phenomenon. To test the hypothesis that a key function of PATJ is to promote preferential adsorption of ZO-1 to the apical interface, we quantified the protein–protein interaction of PATJ with ZO-1 condensates (Fig. 5a–c). Guided by previous work that identified PATJ domains mediating the link to apical complex and the ZO scaffold^24,33^, we investigated the rescue of junctional belt formation and permeability of PATJ truncations in the ∆L27-PATJ tissue (Fig. 5a).

First, we quantified interactions of ZO-1 condensates with PATJ truncations using a cellular partitioning assay^2^ by coexpressing both proteins in HEK293 cells and determined the partitioning coefficient of PATJ in ZO-1 condensates (Fig. 5b,c). We found that full length PATJ partitioned into ZO-1 condensates, indicating attractive interactions. Partitioning of PATJ depended to a large degree on the presence of the PDZ1 and PDZ6 domains. Accordingly, a minimal PATJ construct that contained the apical membrane-binding domain L27 and the PDZ1 and PDZ6 domains showed partitioning. Finally, we used a chimeric construct with the membrane-binding domain of PATJ fused to ZO-1 to force direct interaction of ZO-1 with the apical membrane. As expected, this construct showed the highest partitioning.

To quantify the ability of PATJ truncations to rescue tight-junction formation, we determined the average ZO-1 belt length per cell (Fig. 5d,e) and the barrier function of the belt by measuring the transepithelial permeability to 10k fluorescent dextran in ∆L27-PATJ tissue rescued with the PATJ truncations (Fig. 5f,g). As shown above, both ZO-1 belt length and the permeability barrier were severely compromised in the ∆L27-PATJ tissue compared with WT tissue. Expression of full length PATJ in the ∆L27-PATJ background resulted in full rescue of belt length and the permeability barrier to WT levels (Fig. 5g). Bringing back only the N-terminal L27-PDZ1 domains did not fully rescue belt formation or the permeability barrier. Similarly, PATJ lacking the PDZ6 domain was able to only partially rescue tight-junction formation and barrier function^33,44,45^. However, the expression of the N-terminal apical binding domain together with the tight-junction interaction domain L27-PDZ1PDZ6 resulted in rescue of ZO-1 belt formation and restoration of the permeability barrier. These data suggest that PATJ indeed requires both apical and ZO-1 binding capability to bring ZO-1 to the apical interface and promote formation of continuous tight-junction belts. Accordingly, the expression of the chimeric construct in the ∆L27-PATJ background resulted in rescue of the permeability barrier and ZO-1 belt length to close to WT levels.

Taken together, the results of the rescue experiments support the hypothesis that ZO-1 interacts with the apical membrane through PATJ. The chimeric ZO-1–PATJ construct shows that direct interactions between PATJ and ZO-1 are sufficient to enable extension of ZO-1 condensates along the apical interface.

## Discussion

Our study provides direct evidence that tight-junction belt formation involves a wetting phenomenon of ZO-1 condensates that interact with apical membrane interface. Investigating the compositional changes of junctional condensates during the junction assembly process, we found that apical proteins—in particular, PATJ—come into molecular proximity to ZO-1 during the time when junctional condensates elongate around the apical cell perimeter. Earlier work has described PATJ as a protein of the apical polarity complex that is involved in tight-junction formation^24,39,46^. Our genetic perturbations showed that PATJ is required for elongation of ZO-1 condensates into a functional tight-junction belt (Fig. 2d–f). These results led us to the hypothesis that ZO-1 condensate elongation is causally linked to its interaction with the apical interface.

We speculated that the elongation of nucleated condensates around the apical perimeter could be a wetting phenomenon. Wetting of condensates on membranes requires attractive interactions between the membrane and the condensate^7^, which could be mediated by PATJ. In line with this idea, super-resolution imaging showed that PATJ was enriched at the apical membrane interface of ZO-1 condensates (Fig. 3), and partitioning experiments showed that PATJ interacted with ZO-1 condensates (Fig. 5a–c).

**Extended Data Fig. 7:**
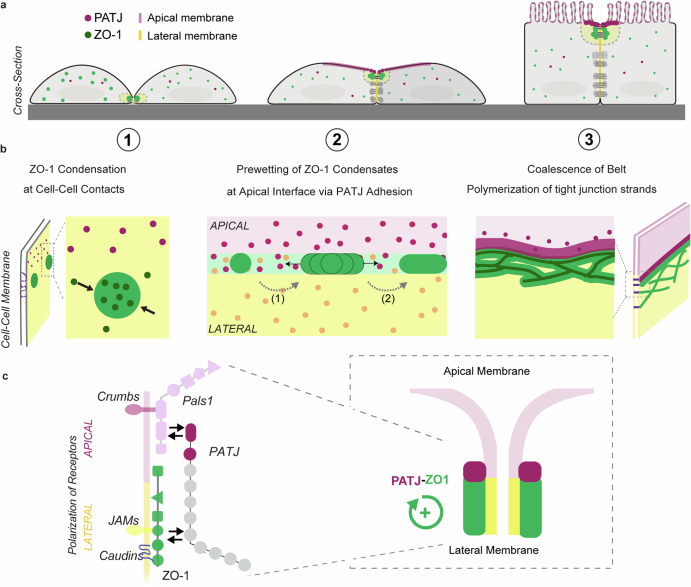
Model of tight junction belt formation by prewetting of junctional condensates along the apical membrane interface. (a) Cross-section view of tight junction formation phases on the cellular level. (1) ZO-1 (green) condensates nucleate at cell-cell contact sites. (2) Cells polarize and nucleated ZO-1 surface condensates grow around the apical interface (magenta). (3) Cell-cell contacts mature, and the tight junction belt closes and seals the tissue. (b) Mesoscale events during tight junction belt formation. (1) Nucleation of ZO-1 condensates leads to partitioning of junction proteins. (2) Interaction of nucleated condensates with the apical interface via PATJ induces condensate growth along the interface via a prewetting transition. Growing condensates fuse into a continuous tight junction belt. (3) Polymerization of tight junction strands establishes the tight trans-epithelial barrier. (c) Molecular interactions underlying prewetting of ZO-1 condensates along the apical interface. ZO-1 surface condensates nucleate by ZO-1 binding to receptors that bind in *trans* at cell-cell adhesion sites (JAMs, Claudins). PATJ binds to the apical membrane via the apical complex (Pals1). Preferential interactions of membrane bound ZO-1 and PATJ mediate enrichment of the ZO-1 – PATJ complex at the apical interface. Apical interface enrichment promotes growth of ZO-1 surface condensates via prewetting.

We compared the elongation dynamics in cells with the thermodynamics of condensate wetting (Supplementary Note 1). This analysis showed that a prewetting transition of ZO-1 along the apical interface recapitulated the key experimental findings, that is, constant growth velocity and mass increase (Fig. 4). In contrast to classical wetting, which describes the spreading of three-dimensional droplets on a surface, prewetting describes the formation of a condensed layer on a surface below the saturation concentration of three-dimensional bulk phase separation^10,47^. On the basis of our results, we propose a model that explains formation of the tight-junction belt as a consequence of a prewetting transition of ZO-1 condensates around the apical–lateral cell perimeter (Extended Data Fig. 7). First, tight-junction formation is initiated by the nucleation of ZO-1 condensates at cell–cell contacts^2^. This leads to formation of isolated surface condensates in the lateral membrane domain that sequester proteins required for assembly of tight junctions. Subsequently, condensates interact with PATJ, which is anchored in the apical membrane through apical receptors^37,48^. Interactions of ZO-1 with the apical–lateral membrane interface drive the growth of condensates along the apical interface into a continuous functional belt through a prewetting transition. Further work is required to determine the detailed molecular interactions between the apical polarity complex around PATJ and ZO-1 condensates.

A nucleation and growth prewetting mechanism of tight-junction belt formation has several implications. It explains the timing, positioning and structure of the tight-junction belt as a consequence of the collective physicochemical properties of the membrane surface and the ZO-1 scaffold. That apical polarity and the junctional complex are connected has been known for a long time^30,45^. However, how this connection relates to the assembly and the structure of the tight-junction belt has remained unclear. A two-step prewetting mechanism of adhesion-mediated ZO-1 surface condensate nucleation and polarity-mediated condensate growth effectively couples both processes to guide the assembly of the mesoscale tight-junction structure in space and time. This prewetting transition of junctional condensates could also explain how the junctional complex can dynamically adapt to shape and length changes of the apical perimeter due to tissue mechanics. Any change in interface length due to stretching or contraction is directly ‘sensed’ by the ZO-1 condensate. In principle, any gaps that form in the ZO-1 belt as a result of mechanical stretching are expected to ‘self-heal’ through the condensate growth mechanism described above.

In addition to condensation and prewetting, belt formation involves active processes such as actin polymerization and claudin strand formation^6,49^. Our data indicate feedback among ZO-1 prewetting, condensate turnover and claudin polymerization (Extended Data Fig. 3j); that is, in ∆L27-PATJ tissues, condensate often shrink after initial nucleation (Extended Data Fig. 6a,b), and fluorescence recovery after photobleaching (FRAP) measurements show that the remaining ZO-1 membrane condensates are less dynamic than those in WT tissue (Extended Data Fig. 6c–e). A similar reduction in ZO-1 dynamics has previously been observed after blocking actin polymerization^50^. Together, these observations suggest that prewetting of ZO-1 condensates feeds back to active processes at the apical interface. How the collective properties of the junctional condensates provide structural flexibility and robustness with respect to cell shape changes during development will be an exciting avenue for future studies.

More generally, our work has important implications for how cells exploit the collective physical properties of protein condensates to actively shape higher-order structures. Recent examples have shown that interactions of protein condensates with biological surfaces such as DNA and RNA, cytoskeletal filaments or lipid membranes can drive mesoscale shape changes such as DNA compaction, filament bundling and branching, and membrane budding and wrapping^7,12,15,47,51^. The common theme that emerges from these different examples is that shape changes are driven by forces arising at the interface of protein condensates with cellular structures. Here we uncovered a striking example of how cells use the polarized organization of the epithelial cell membrane to position and shape junctional condensates via a prewetting transition. We expect that the emergent properties of protein condensates will serve as a general example of how complex mesoscale structures self-assemble in cells. Our findings also provide a new perspective on how to manipulate tight-junction permeability in biomedical applications.

## Methods

### Cell culture in two-dimensional monolayers

HEK293 and MDCK-II (00062107, Public Health England) cells were cultured as two-dimensional monolayers in minimal essential medium with 5% fetal bovine serum, 1% non-essential amino acids, 1% sodium pyruvate, and 1% GlutaMAX without addition of antibiotics at 37 °C with 5% CO_2_. Two-dimensional monolayers on transgenic cell lines were selected in the presence of geneticin (400 μg ml^−1^). Transient transfection of MDCK-II cells was carried out using Lipofectamine 2000 after cells reached a confluency of 70%. Transgenic cell lines were created from synthesized genes cloned using Not-I and Asc-I cutting sites into mammalian expression plasmids designed in-house (pOCC series) with N-terminal Dendra2, a selection marker against neomycin-geneticin and a CMV promotor. To obtain stable lines, single clone selection was carried out by fluorescence-activated cell sorting.

For seeding of two-dimensional monolayers, 400 µl of cell suspension (0.5 million cells per millilitre) was transferred on to the external side of a standing transwell filter (Corning 3460). Cells were left to adhere at 37 °C in a sterile environment for 30 min. Afterwards, each transwell filter was mounted in a one-well plate with 1 ml of culture medium on the bottom side and 500 µl of medium on the internal side.

### Generation of three-dimensional cysts and transepithelial permeability assay

For the adherent three-dimensional cell culture cysts, MDCK-II cells were resuspended from a confluent monolayer into a single-cell suspension. The surface of a Mateck dish (35 mm glass bottom, P35G-0.170-14-C) was coated with a solution of laminin (0.5 mg ml^−1^) for 1 h at 37 °C, 5% CO_2_. Afterwards, a suspension of 20,000 cells were seeded on the coated surface in the respective culture medium complemented with 5% Matrigel on ice. Cells were cultured for 5–6 days until reaching 30–40 µm in diameter. To measure the permeability of dextran (Alexa647, 10k) cyst medium was supplemented with 10 μM dextran. After 15 min of incubation, cysts were imaged using a confocal microscope to evaluate the distribution of dextran. The permeability of dextran into the lumen was quantified by measuring the intensity in the lumen and dividing this by the average intensity outside the cysts.

### CRISPR–Cas9 knock-in MDCK-II cells

CRISPR–Cas9 technology was used to generate several single and double knock-in MDCK-II cell lines. ZO-1–mNeon was generated by fusing a copy of mNeon at the N terminus of the initial exon of endogenous ZO-1. Using the cell line expressing this fusion construct as a background, we tagged several other scaffold proteins with mScarlet at the N terminus of the initial exon of endogenous PATJ or ZO-2 and at the carboxyl terminus of MAGI-3 to generate double knock-in lines. Briefly, specific CRISPR RNA (crRNA, Integrated DNA Technologies (IDT) lt-R CRISPR–Cas9 crRNA) for each gene of interest was designed using online tools Crispor (http://crispor.tefor.net) and ChopChop (https://chopchop.cbu.uib.no). *Trans*-activating crRNA (IDT catalogue no. 1072532) and crRNA were annealed at a ratio of 1:1 by incubation for 5 min at 95 °C and then 10 min at room temperature to generate guide RNA (gRNA). All gRNAs can be found in Supplementary Table 4. Next, the donor plasmid containing 5′ and 3′ homology arms was synthesized in a pUC57 Kan (Genescript) or a pUCIDT Kan (IDT) (Supplementary Table 4). A PTisy plasmid containing the fluorescence tag (mSc or mNn) was integrated into the donor plasmid through digestion using restriction enzymes. Afterwards, a ribonucleoprotein complex was assembled by mixing 1 μl (10 μg μl^−1^) of the recombinant Alt-R S.p. HiFi Cas9 nuclease (IDT, catalogue no. 1081060) and 1 μl of gRNA (100 μM) with the reaction buffer, followed by incubation at room temperature for 20 min. The ribonucleoprotein complex and 1 μg of the donor plasmid were cotransfected through electroporation and overlapped with the exon sides. Electroporation of each complex was performed in 300,000 cells (Invitrogen NEON electroporation machine and kit, two pulses, 20 ms, 1,200 V). Cells were then plated in growth medium in a six-well plate. Medium was exchanged after 24 h. Cells were sorted 48–72 h after electroporation. Fluorescent cells (mNn or mSc) were enriched (one or two cycles), and single clones were plated in one well of a 96-well plate. To validate genetic modification, we first did a PCR amplification. The correct insertion of the fluorescent tag at the correct locus of the gene of interest was verified by sequencing genotyping. Sequencing confirmed homozygous insertion of the tags, and imaging confirmed that mS tagging of the endogenous proteins resulted in proper colocalization with mN-ZO-1 at the tight-junction belt in confluent monolayers.

### CRISPR–Cas9 knockout in MDCK-II cells

For deletion of PATJ ∆exon 3, we used two gRNAs on each site of the targeting exon for each gene, selected on the basis of low off-target activity using http://crispor.tefor.net. The gRNAs were ordered as crRNAs from IDT. gRNAs were transfected in pairs, and cell pools were tested for deletion events by PCR using primers spanning the targeting exon. Best-performing gRNA pairs were used for cell cloning. Single cells were screened for the deletion by PCR using primers spanning the targeting exon. Flanking PCR with one primer outside and one primer inside the deletion was performed on the knockout candidate clones to verify the absence of the WT allele. Deletion alleles were verified by Sanger sequencing.

Sequencing of two clones confirmed deletion of the first exon, including a frame shift leading to an early stop codon. Western blots and quantitative PCR (qPCR) against the N-terminal L27 domain of PATJ, encoded by the first exon, confirmed deletion of this domain. Immunostaining of the more C-terminal PDZ4 confirmed that the remaining truncated protein was located in the cytosol and was not present at the tight junction. qPCR was performed using a Qiagen RNeasy (74104) mini kit after bioanalysis showed that the primers were of good quality (Supplementary Table 4).

### Two-colour super-resolution STED microscopy

STED imaging was performed with a commercial confocal infinty-line STED microscope (Abberior Instruments) operated using the Imspector software (v.16.2.8415), with pulsed laser excitation (490 nm, 560 nm, 640 nm, 40 MHz), and ×60 water and ×100 oil objectives (Olympus). Star Orange was imaged with a pulsed laser at 560 nm, and excitation of Abberior Star Red was performed at 640 nm. The depletion laser for both colours was a 775 nm, 40 MHz, pulsed laser (Katana HP, 3 W, 1 ns pulse duration, NKT Photonics). The optimal combination of excitation intensity, STED power and pixel dwell time were established by minimizing the onset of strong photobleaching. To reduce high-frequency noise, STED images were filtered with a two- or three-dimensional Gaussian with a sigma of 0.8 pixels. Quantification and segmentation of STED data was done using custom software written in MATLAB^52^.

### Live imaging microscopy

Live imaging kinetics were acquired using a Wide-Field Delta Vision Elite system equipped with an Olympus PlanApo N ×60/1.42 objective, SSI Lumencor illumination system and Roper Evolve EMCCD camera. Perturbation experiments were acquired with a wide-field General Electric Delta Vision system equipped with an Olympus PlanApo N ×60/1.42 objective, and mN and mCh were excited with an Applied Precision Xenon Arc Lamp (V300-Y18). High-resolution imaging was done with a Yokogawa Spinning Disk Field Scanning Confocal System (CSU-X1 Nikon, Andor iXOn Ultra) using a ×60 water objective.

### MDCK-II calcium switch assay

The calcium depletion experiments were performed on a confluent monolayer of MDCK-II with different cell lines. Cells were grown for 18 h in calcium-free media until tight junctions were disrupted. For proteomics experiments, medium containing calcium was added to the cells at 37 °C, 5% CO_2_, and each sample was prepared at 0 h, 0.5 h, 1 h, 3 h, and 18 h afterwards. For live imaging experiments, medium containing calcium was added to the cells directly on the microscope chamber at 37 °C, 5% CO_2_, and tight-junction formation was imaged every 1 min for 3 h or every 30 min for 18 h.

### Transepithelial resistance

MDCK-II cells were seeded into a Corning transwell plate with membrane inserts (12 mm, 0.4 mm pore), and transepithelial resistance was measured at various time points from 1 h after seeding until 6 days. Measurements were acquired using a two-electrode resistance system from Millipore (Millicell ERS-2) inserted between the apical and basal parts of the chamber. The electric resistance was multiplied by the growth area of the transwell filters (1.12 cm^2^), and the resistance of the medium without cells was subtracted to give a final result in Ω × cm^2^.

### Quantification of ZO-1 belt length and cell perimeter coverage

To determine the coverage of the cell perimeter by ZO-1 (%) or the ZO-1 belt length per cell (µm per cell), we performed the following steps. We segmented the cells in the monolayer with CellPose^53^ using the bright-field channel as input. The outline of the CellPose segmentation was used as the cell perimeter length. We segmented the mN-ZO-1 channel through local intensity thresholding and skeletonized the segmented image to obtain the length of the ZO-1 belt using FIJI. The percentage of the cell perimeter covered by ZO-1 was calculated by the following formula: perimeter coverage = (TJ length/cell periphery) × 100%. The length of the ZO-1 belt per cell, *L*_ZO-1_ (in μm per cell), was calculated using MATLAB as follows: *L*_ZO-1_ = sum(skeletonized ZO-1 image)/cell number.

### Determination of tight-junction protein recruitment kinetics

To determine the *t*_1/2_ arrival kinetics of the mS-tagged tight-junction proteins from the two-colour time series, we segmented the condensed mN-ZO-1 signal and quantified the mScarlet intensity in the segmented ZO-1 condensates and the cytoplasm over time. Cell segmentation was done using CellPose (https://github.com/mouseland/cellpose), and ZO-1 condensate segmentation used the plugins Subtract Background and Make Binary in FIJI. To directly correct for photobleaching artefacts, we calculated the ratio between the junctional and the cytoplasmic mScarlet signal for each time point using custom MATLAB code^52^. Assuming that bleaching is spatially homogenous, this ratio is independent of bleaching and directly reports the enrichment of the protein in the condensed ZO-1 (tight junction). Kinetic data were fitted using a HiII slope model in Prism with a nonlinear fit variable slope (four parameters) to calculate the half-maximal effective concentration of each protein. To calculate the *t*_1/2_ values for client protein arrival, we fitted the kinetic data to a Hill slope model and determined the difference in arrival time between ZO-1 and the client protein in living cells.

### Determination of ZO-1 condensate extension and eccentricity

ZO-1 condensate eccentricity was quantified on segmented ZO-1 images using the function regionprops to measure the eccentricity of segmented condensates over time in MATLAB. The eccentricity is the ratio of the distance between the focus of the ellipse and its major axis length; it takes a value between 0 and 1. (Here, 0 and 1 are degenerate cases; an ellipse whose eccentricity is 0 is a circle, whereas an ellipse whose eccentricity is 1 is a line segment.)

### Extension and intensity rates

To determine the extension of the condensates over time, we used the plugin JFilament in FIJI^54^. The analysis directly provided the length of the condensates over time. To quantify the amount of ZO-1 material in the condensate over time, we used the segmentation of the JFilament tracks to measure the sum intensity of the condensate per time point in MATLAB. As the JFilament tracks were one-dimensional, we extended the width of the segmentation to fit the width of the condensate.

### Fluorescence recovery after photobleaching

FRAP experiments in cells were carried out with the following settings on a confocal infinty-line STED microscope (Abberior Instruments). The region of interest was bleached using a 405 nm diode at 1.5 mW at the back focal plane of the objective, with 100 ms pixel dwell time. Prebleaching and postbleaching images were acquired using a 490 nm laser at 5 μW. Fluorescence recovery of mNeon was monitored for 1–20 min with a time resolution of 11 s. Cell movements during the recovery were corrected by registration of all frames to the first frame using the plugin StackReg in FIJI.

### Client partitioning assays in HEK293

HEK293 cells were transfected with ZO-1–GFP, and clients were tagged with mCherry. We measured the fluorescence intensity of the client inside and outside the condensate of ZO-1, and the background outside the cell was subtracted from those values.

### Immunoblotting and in-gel fluorescence

Aliquots of approximately 5 μg of input, elution and beads in lysate buffer (10% glycerol, 2% sodium dodecyl sulfate (SDS), 1 mM dithiothreitol (DTT), 1x Protease Inhibitor Cocktail, 50 mM Tris-HCl, pH 8) were subjected to SDS polyacrylamide gel electrophoresis (4–20% Tris-Glycine Novex gels) at 90 mV for 3 h. An iBlot2 gel transfer system (Thermo Fisher, IB21001) was used to transfer proteins on to a nitrocellulose membrane (10 min, 20 V). An iBind Flex system (Thermo Fisher, SLF2000) was used to detect protein levels by immunoblotting, with the following antibodies: IRDye-800CW streptavidin (1:4000, LI-COR, 92632230). Membranes were scanned with an LI-COR Odyssey system at 700 nm and 800 nm. In-gel fluorescence of the tagged proteins (Dendra2) was done in a Typhoon FLA 7000 scan system (GE). For western blot detection of PATJ, a wet system was used for transfer (260 mA for 2 h), and classical HRP detection of proteins was done using superSignal West Pico Plus chemiluminescent substrate (Thermo Fisher, catalogue no. 34580) on GE hyperfilms (Cytiva, 28-9068-50). Primary antibody incubation was performed overnight at 4 °C in 5% milk and 0.1% Tween, with PATJ-L27 (rabbit polyclonal, gifted by Le Bivic’s lab) 1:200, PATJ-PDZ4 (rabbit polyclonal, LSBio, LC-C410011), and 1:500 β-actin (rabbit polyclonal, Abcam, ab8227). The following secondary antibodies were used at 1:5000 dilution: goat anti-rabbit IgG-HRP (H+L) (Cell Signaling, catalogue no. 7074), and goat anti-mouse IgG-HRP (H+L) (Cell Signaling, catalogue no. 7076) (Supplementary Fig. 1).

### Immunofluorescence

Fixation was performed in the same way for both two-dimensional monolayers and three-dimensional cysts. Cells were fixed with 4% paraformaldehyde in phosphate-buffered saline (PBS) for 10 min at room temperature, followed by quenching in 300 mM glycine, and permeabilized with 0.5% Triton X-100 in PBS for 10 min. Cells were blocked with 2% bovine serum albumin and 0.1% Triton X-100 in PBS for 1 h at room temperature. Staining of all primary and secondary antibodies involved incubation at room temperature for 2 h or 30 min with a dilution of 1:50 or 1:200, respectively, in blocking buffer. Staining for neutravidin-647 (neutravidin, A-2666 and Alexa Fluor 647 succinimidyl ester A-20006, Invitrogen) was performed at 1:1000 dilution for 1 h at room temperature in blocking buffer. Primary antibodies were PATJ-L27 rabbit polyclonal (produced in-house), PATJ-PDZ4 rabbit polyclonal (LSBio, LC-C410011), PALS1 mouse monoclonal (Santa Cruz, sc-365411), ZO-1 mouse monoclonal IgG1 (Invitrogen, 33-9100), occludin rabbit polyclonal (Life Technologies, 71-1500), Lin7 rabbit polyclonal (Thermo Fisher, 51-5600), E-cadherin rabbit IgG, (Cell Signalling, 3195S). The secondary antibodies were goat anti-mouse (Abberior, star-red 2-0002-011-2) and goat anti-rabbit star-orange (Abberior, storange-1102)

### APEX2 labelling during tight-junction formation

Cells were plated in a T75 flask after reaching confluency, and the full medium was substituted with medium without calcium for 18 h to enable the cells to reach a rounded, non-polarized state. Afterwards, the medium was replaced with full medium again, and APEX2 labelling was performed at various time points by adding 1 mM biotin phenol to the cells for 30 min before the addition of 1 mM H_2_O_2_ for 1 min. Immediately after, the reaction was quenched on ice by washing three times with 1× PBS supplemented with 10 mM sodium ascorbate (Sigma A4034), 10 mM sodium azide, and 5 mM Trolox ((+/−)-6-hydroxy-2,5,7,8-tetramethylchromane-2-carboxylic acid, Sigma 238813). To validate biotinylation through fluorescence microscopy, cells were immediately fixed after this step. For proteomics and western blot analyses, cells were lysed by scraping them off the growth surface into ice-cold lysis buffer (2% SDS, 10% glycerol, 1 mM DTT, 50 mM Tris-HCl, pH 8, 1× Protease Inhibitor Cocktail Set III EDTA-Free (EMD Millipore, catalogue no. 539134), supplemented with 10 mM sodium ascorbate, 10 mM sodium azide and 5 mM Trolox. The lysate in was collected in a low-protein-binding reaction tube and 5 μl of benzonase was added, followed by incubation in a shaker for 15 min at 37 °C, 700 rpm. Finally, 1 mM EDTA and 1 mM EGTA were added, and the sample was spun down using a table centrifuge to remove debris. After lysis, the protein concentration was checked using a Pierce 660-nm (Thermo Fisher Scientific, catalogue no. 22660) protein assay test before pull-down to ensure the same starting protein concentration was used. Lysates were aliquoted into 1,500 μg of total protein, snap frozen and stored at −80 °C.

### Streptavidin pull-down of APEX2 biotinylated proteins

All buffers used for pull-down proteomics experiments were freshly made and filtered with a 0.22 μm filter before use. Frozen lysates (1,500 g protein) were diluted 1:10 in 50 mM Tris, pH 8, and placed in an incubation buffer consisting of 0.2% SDS, 1% glycerol, 1 mM DTT, 1 mM EGTA, 1 mM EDTA in 50 mM Tris-HCl, pH 8, and 1× protease inhibitor. For affinity purification, approximately 100 l of streptavidin magnetic beads (Pierce, PI88817) were washed in incubation buffer two times before binding. Beads were added to the sample in a total volume of 2 ml and incubated for 2 h at room temperature in a rotating wheel. Next, beads were pelleted down using a magnetic rack, and the supernatant was collected and kept for western blot analysis. Each sample of beads containing the bound biotinylated proteins was washed with a series of ice-cold buffers (2 ml each) to remove unspecific binders. The beads were then washed twice with washing buffer (0.2% SDS, 1% glycerol, 1 mM DTT, 1 mM EGTA, 1 mM EDTA in 50 mM Tris-HCl pH 8, 1× protease inhibitor), once with 1 M KCl, once with 2 M urea in 50 mM Tris-HCl pH 8, once with 2 mM biotin 50 mM Tris-HCl pH 8, and finally three times with 50 mM Tris-HCl pH 8. Biotinylated proteins were eluted by boiling the beads in 50 μl of elution buffer (5% SDS, 10% glycerol, 20 mM DTT, in 50 mM Tris-HCl, pH 8, 1× protease inhibitor) at 95 °C for 10 min, followed by cooling on ice and a brief spin-down. Samples were placed on a magnetic rack, and the eluate was collected in a new tube for proteomics and SP3. Magnetic beads and 10 μl of eluate were together subjected to western blotting for validation of the biotinylation experiments before mass spectrometry.

To process all time points in the same quantitative mass spectrometry analysis, we used a multiplex proteomic approach based on tandem mass tag (TMT). The TMT isobaric tagging approach enables robust quantitative proteomics by measuring all samples in one run. This enabled statistical analysis of relative protein enrichment at different time points after calcium switch across proteins detected at all time points.

### Mass spectrometry and TMT labelling

Reduction of cysteine-containing proteins was performed with dithiothreitol (56 °C, 30 min, 10 mM in 50 mM HEPES, pH 8.5). Reduced cysteines were alkylated with 2-chloroacetamide (room temperature, in the dark, 30 min, 20 mM in 50 mM HEPES, pH 8.5). Samples were prepared using the SP3 protocol^55,56^, and 300 ng trypsin (sequencing grade, Promega) was added per sample for overnight digestion at 37 °C. Peptides were labelled with TMT10plex Isobaric Label Reagent (Thermo Fisher) according to the manufacturer’s instructions. In short, 0.8 mg reagent was dissolved in 42 μl acetonitrile (100%), and 8 μl of stock solution was added, followed by incubation for 1 h and quenching with 5% hydroxylamine for 15 min at room temperature. Samples were combined for the TMT10plex, and an OASIS HLB µElution Plate (Waters) was used for further sample clean-up^57^. Offline high-pH reverse-phase fractionation was performed using an Agilent 1200 Infinity high-performance liquid chromatography system equipped with a quaternary pump, degasser, variable-wavelength ultraviolet detector (254 nm), and Peltier-cooled autosampler and fraction collector (both set at 10 °C for all samples).

The column was a Gemini C18 column (3 μm, 110 Å, 100 × 1.0 mm, Phenomenex) with a Gemini C18, 4 × 2.0 mm SecurityGuard (Phenomenex) cartridge as a guard column. The solvent system consisted of 20 mM ammonium formate (pH 10.0) (phase A) and 100% acetonitrile as the mobile phase (B). The separation was accomplished at a mobile phase flow rate of 0.1 ml min^−1^ using the following linear gradient: 100% A for 2 min, from 100% A to 35% B in 59 min, to 85% B in a further 1 min, and held at 85% B for 15 min, before returning to 100% A and re-equilibration for 13 min. Thirty-two fractions were collected during liquid chromatography separation and subsequently pooled into six fractions. The first and the two last fractions of the 32 were discarded and not used at all. Pooled fractions were dried under vacuum centrifugation and reconstituted in 15 μl 1% formic acid, 4% acetonitrile, for liquid chromatography coupled with tandem mass spectrometry analysis.

### Mass spectrometry data acquisition

Peptides were separated using an UltiMate 3000 RSLC nano liquid chromatography system (Dionex) fitted with a trapping cartridge (µ-Precolumn C18 PepMap 100, 5 µm, 300 µm i.d. × 5 mm, 100 Å) and an analytical column (nanoEase M/Z HSS T3 column 75 µm × 250 mm C18, 1.8 µm, 100 Å, Waters). Trapping was carried out with a constant flow of solvent A (3% dimethyl sulfoxide, 0.1% formic acid in water) at 30 µl min^−1^ on to the trapping column for 6 min. Subsequently, peptides were eluted through the analytical column with a constant flow of 0.3 µl min^−1^ with an percentage of solvent B (3% dimethyl sulfoxide, 0.1% formic acid in acetonitrile) increasing from 2% to 8% in 4 min, from 8% to 28% in 104 min, from 28% to 40% for a further 4 min and finally from 40% to 80% for 4 min before returning to the equilibration condition of 2%. The outlet of the analytical column was coupled directly to an Orbitrap Fusion Lumos Tribid mass spectrometer (Thermo Fisher) using the proxeon nanoflow source in positive ion mode.

The peptides were introduced into the Fusion Lumos through a Pico-Tip Emitter 360 µm OD × 20 µm ID 10 µm tip (New Objective) with an applied spray voltage of 2.2 kV. The capillary temperature was set to 275 °C. A full mass scan was acquired with a mass range of 375–1500 *m*/*z* in profile mode in the Orbitrap with a resolution of 120,000. The filling time was set to a maximum of 50 ms with a limitation of 4 × 10^5^ ions. Data-dependent acquisition was performed with the resolution of the Orbitrap set to 30,000, a fill time of 94 ms and a limitation of 1 × 10^5^ ions. A normalized collision energy of 36 was applied. MS2 data were acquired in profile mode.

### Mass spectrometry analysis

IsobarQuant and Mascot (v.2.2.07) were used to process the acquired data. The data were then searched against the UniProt *Canis lupus* proteome database (UP000805418), which contains common contaminants and reversed sequences. The following modifications were included in the search parameters: carbamidomethyl (C) and TMT10 (K) (fixed modifications); and acetyl (N-term), oxidation (M), and TMT10 (N-term) (variable modifications). A mass error tolerance of 10 ppm and 0.02 Da was set for the full scan (MS1) and the MS/MS spectra, respectively. A maximum of two missed cleavages was allowed, and the minimum peptide length was set to seven amino acids. At least two unique peptides were required for protein identification. The false discovery rate (FDR) at the peptide and protein level was set to 0.01. The R programming language (ISBN 3-900051-07-0) was used to analyse the raw output data of IsobarQuant. Potential batch effects were removed using the limma package. Variance stabilization normalization was applied to the raw data using the vsn package. Individual normalization coefficients were estimated for different time points compared with the non-calcium condition (*t*_0_). Normalized data were tested for differential expression using the limma package. The replicate factor was included in the linear model. For comparisons of different time points versus the *t*_0_ condition, proteins with fold change greater than 2 were considered to be potential hits, and an FDR threshold of 0.05 was used to filter out noisy data. In addition, we excluded false positive hits due to non-junctional interactions (ribosomes, nucleus, mitochondria, endoplasmic reticulum (Supplementary Table 2).

In experiments comparing different time points, proteins were first tested for their enrichment compared with a −*t*_0_ control. R package fdrtool35 was used to calculate FDRs using the *t* values from the limma output. Proteins with an FDR less than 5% and a consistent fold change of at least 10% in each replicate were defined as hits. The ggplot2 R package was used to generate graphics. Proteins matching a false positive list of non-biotinylated proteins were removed (Supplementary Table 3). RStudio code was adapted from previously reported code (https://github.com/fstein/EcoliTPP). R packages used were limma (https://bioconductor.org/packages/limma), MSnbase (https://bioconductor.org/packages/MSnbase), tidyverse (https://tidyverse.tidyverse.org), biobroom (https://bioconductor.org/packages/biobroom), ggrepel (https://cran.r-project.org/web/packages/ggrepel/vignettes/ggrepel.html) and ClusterProfiler (https://bioconductor.org/packages/clusterProfiler/). The interactome was created in Cytoscape (v.3.9.0) using the STRING database (v.11.5). Cell localization gene ontology annotations were from http://geneontology.org. The mass spectrometry proteomics data have been deposited at the ProteomeXchange Consortium through the PRIDE^58^ partner repository with dataset identifier PXD052221.

### Quantification and statistical analysis

Images were analysed with FIJI (https://fiji.sc/) and MATLAB (MathWorks). All data are expressed as the mean ± s.d., mean ± s.e.m. or mean ± 95% confidence interval, as stated in the figure legends and results. Values of *n* and what *n* represents (for instance, number of images, condensates or experimental replicates) are stated in figure legends and results. Two-tailed Student’s *t*-test or one-way ANOVA was used for normally distributed data. Statistical analyses used Kruskal–Wallis test with post hoc Dunn’s multiple-comparison test.

### Numerical calculations for the thermodynamic model

Numerical calculations were done using programming language Python v.3.8.10; all code was run using IPython v.7.3.10. This software comes preinstalled in most of the Linux distributions. OS: Ubuntu 20.04.6 LTS, 64-bit, GNOME v.3.36.8. All code and a minimal dataset are available at Zenodo (https://zenodo.org/doi/10.5281/zenodo.11174400)^52^.

### Reporting summary

Further information on research design is available in the Nature Portfolio Reporting Summary linked to this article.

## Online content

Any methods, additional references, Nature Portfolio reporting summaries, source data, extended data, supplementary information, acknowledgements, peer review information; details of author contributions and competing interests; and statements of data and code availability are available at 10.1038/s41586-024-07726-0.

### Supplementary information

Supplementary Information Supplementary Notes 1–4 and references.

Reporting Summary

Supplementary Figure 1 Uncropped western blots.

Supplementary Table 1 Time-resolved proximity proteomics of tight-junction assembly.

Supplementary Table 2 Heat map of a tight junction over time. Table contains the protein hits shown in the heat map in Fig. 1d, and values displayed are the normalized log_2_ fold change of tight junction proteins at different time points.

Supplementary Table 3 Full dataset for time-resolved proximity proteomics of the tight junction.

Supplementary Table 4 Reagents used in this study. Each tab contains plasmids for mammalian expression, gRNA for CRISPR–Cas-9 knock-in and knockout and primers used for qPCR.

Supplementary Video 1 First hour of mN-ZO1 in WT and ∆L27-PATJ tissue during tight junction formation. Live imaging of mN-ZO-1 knock-in on WT and ∆L27-PATJ MDCK-II during calcium switch assay of the tight junction belt formation. Scale bar, 10 µm, time stamp (h:min).

Supplementary Video 2 mS-PATJ recruitment to mN-ZO-1 condensates at the cell–cell interface. Live imaging of mN-ZO-1 and mS-PATJ dual knock-in on WT MDCK-II during calcium switch assay during tight junction formation. Scale bar, 1 µm, time stamp, h:min.

Supplementary Video 3 mZO-1 membrane condensate extension at cell–cell interface in WT and ∆L27-PATJ. Live imaging of mN-ZO-1 knock-in forming membrane condensate that elongates at the cell–cell interface. Scale bar, 1 µm, time stamp, h:min.

Supplementary Video 4Numerical simulation for single condensate extension analysis. Numerical solutions to the surface condensation theory for two different values of the relative binding affinity $\Delta \bar{\epsilon }$ of ZO-1 to the interface. Top video shows dynamics for $\Delta \bar{\epsilon }=0.43$, and the bottom row shows the case where $\,\Delta \bar{\epsilon }=0.19$.

Supplementary Video 5Numerical simulation for multiple condensate extension analysis. Numerical solutions to the concentration dynamics equation. The top row shows numerical solutions for relative binding affinity $\Delta \bar{{\epsilon }}=0.43$, for which linear extension with time is observed. The bottom row shows numerical solutions for $\Delta \bar{{\epsilon }}=0.19$, for which no extension is observed.

### Source data

Source Data Fig. 2

Source Data Fig. 3

Source Data Fig. 4

Source Data Fig. 5

Source Data Extended Data Fig. 1

Source Data Extended Data Fig. 2

Source Data Extended Data Fig. 3

Source Data Extended Data Fig. 5

Source Data Extended Data Fig. 6

## Data Availability

The article includes all datasets generated or analysed during this study. The code produced for this paper, including STED analysis, recruitment kinetics and numerical calculations for the thermodynamic model, is available with a minimal dataset at Zenodo (https://zenodo.org/doi/10.5281/zenodo.11174400)^52^. Mass spectrometry data were analysed using the UniProt *C. lupus* proteome database (UP000805418). The mass spectrometry proteomics data have been deposited at the ProteomeXchange Consortium through the PRIDE partner repository with dataset identifier PXD052221. Requests for further information or resources and reagents should be directed to A.H. (alf.honigmann@tu-dresden.de). Source data are provided with this paper.

## Code availability

All source code custom-generated for this study are available at Zenodo (https://zenodo.org/doi/10.5281/zenodo.11174400)^52^. This includes the imaging analysis for the STED data, the live imaging segmentation kinetics and the numerical calculations.
