# Supplementary material for: Membrane prewetting by condensates promotes tight-junction belt formation
Source: Nature. 2024 Aug 7;632(8025):647–55. doi: 10.1038/s41586-024-07726-0 (PMC11324514; doi:10.1038/s41586-024-07726-0)
Supplement: Supplementary file 1 — Supplementary Notes 1–4 and references. [file 41586_2024_7726_MOESM1_ESM.pdf]

nature portfolio

https://doi.org/10.1038/s41586-024-07726-0

Supplementary information

Membrane prewetting by condensates promotes tight-junction belt formation

In the format provided by the authors and unedited

Supplementary information

Membrane prewetting by condensates promotes tight junction belt formation

Karina Pombo-García[1], Omar Adame-Arana[2], Cecilie Martin-Lemaitre[1], Frank Jülicher[2,3,5], Alf Honigmann[1,4,5]

Corresponding authors: Karina.pombo-garcia@mpi-cbg.de and alf.honigmann@tu-dresden.de

1 Max Planck Institute of Molecular Cell Biology and Genetics, Dresden, Germany

2 Max Planck Institute for the Physics of Complex Systems, Dresden, Germany

3 Center for Systems Biology Dresden (CSBD), Dresden, Germany

4 Technische Universität Dresden, Biotechnologisches Zentrum, Center for Molecular and Cellular Bioengineering (CMCB), Dresden, Germany

5 Cluster of Excellence Physics of Life, TU Dresden, Dresden, Germany

Supplementary Note 1. Thermodynamic theory of junctional condensates growth

Binding, wetting, prewetting and their possible relation to ZO-1 surface condensates growth

Here we compare different plausible physical mechanisms that could drive the experimentally observed linear growth of ZO-1 surface condensates around the apical interface. First, we consider the scenario in which simple binding would give rise to the enrichment of ZO-1 at the apical interface. In this scenario, ZO-1 proteins would bind with a high affinity to a substrate that is located at the apical interface, presumably PATJ. Simple binding would lead to a homogenous enrichment of ZO-1 at the apical interface instead of something reflecting growth starting from a condensed phase. This scenario does not reflect the experimentally observed linear growth of ZO-1 condensates, pointing to a different physical mechanism of growth.

As a second possible scenario we consider condensate wetting dynamics, which are characterized by the motion of the triple line. In this case, pre-formed 3D ZO-1 droplets would wet the apical interface and spread via capillary forces. This scenario predicts that condensates extension slows down over time with $E(t)\sim t^{1/10}$ in the case of a 3D bulk condensate, or with $E(t)\sim t^{1/7}$ in the case of a surface condensate (2D) [1,2]. In cells the extension of ZO-1 condensates varies linearly with time implying that junctional condensates grow with constant speed (Fig.4b). Furthermore, condensate growth in cells is associated with the addition of material that is recruited from the bulk (Fig.4c), in contrast to 3D condensates spreading on a surface where material stays constant. We could therefore rule out spreading via triple line dynamics or capillary motion[2,3]. In addition, a classical wetting scenario would require that the cytosolic concentration of ZO-1 is above its saturation concentration for bulk phase separation, which is not the case. Instead, ZO-1 concentration in the cytoplasm is around 700 nM, which is far below the saturation concentration for 3D phase separation in cells (around 12 μM)[4]. Thus, the system is in a sub-saturated regime, which suggests the possibility of a prewetting transition[5].

A prewetting transition is a type of surface phase transition below the saturation concentration for 3D phase separation, which leads to the formation of a condensed layer on the surface (Fig.4f, Extended Data Fig. 5d (up). As we show below the scaling of surface condensate growth in a prewetting scenario reproduces the observed linear growth $E(t)\sim t$. In addition, structural and dynamical evidence suggests that junctional ZO-1 condensates are indeed organized as a condensed surface layer[6] and FRAP measurements (Extended Data Fig 6 c-e) show exchange of condensate material with two distinct kinetics, suggesting a more tightly bound first layer and more loosely bound additional layers[4,7]. Below we discuss a minimal model that recapitulates the experimental observations and provide a thermodynamic basis for the formation of the tight junctional belt.

Below we describe the thermodynamics of tight junction belt formation as a two-step process: First condensate nucleation at adhesion sites, second condensate growth around the apical interface. Both nucleation and growth can only happen at the membrane surface, because the system is in a prewetting regime. Nucleation is promoted by membrane binding of ZO-1 to adhesion receptors. The initial size of the nucleated surface condensate is limited to the adhesion sites. Condensate growth around the apical membrane perimeter is then promoted by ZO-1 condensate interactions with apical PATJ. The condensates grow along the apical interface by recruiting additional molecules from the bulk via a prewetting transition.

Thermodynamics of ZO-1 condensation on a membrane

We describe a membrane that is polarized into a lateral and apical domain (Fig. 4d) and is in contact with a bulk solution containing ZO-1 protein with bulk chemical potential μ_{bulk} (Fig. 4f and Extended Data Figure 5d). ZO-1 proteins can bind to the membrane, where they can form condensates. The surface density of proteins is denoted $\rho(\vec{x})$, where $\vec{x}$ is the position on the membrane. We also introduce a dimensionless composition variable $\phi(\vec{x})$, describing surface condensation, where $\phi = \alpha(\rho - \rho_0)$. Here α is a calibration factor and ρ_0 a reference surface density. The thermodynamics of ZO-1 condensation on the membrane is described by the free energy

$$F = \int_S dS\ f(\phi)\ , \qquad (1)$$

where the integral is over the membrane surface S and the free energy density is given by

$$f = \frac{1}{\sigma}\left(-\frac{r}{2}\phi^2 + \frac{\lambda}{4}\phi^4 - \epsilon\phi + \frac{\kappa}{2}(\nabla\phi)^2\right)\ , \qquad (2)$$

here, r and λ are parameters that control the double well shape of the free energy, and σ is the surface area occupied by a single ZO-1 protein molecule on the membrane. The two wells represent dilute and condensed phases of ZO-1 (Fig. 4e). The parameter κ describes a free energy contribution related to interfacial tension and the integration is carried over the membrane surface area. Finally, $\epsilon(\vec{x})$, is the binding affinity of ZO-1 molecules to the membrane, which depends on position $\vec{x}$ on the membrane. ZO-1 molecules bind preferentially to the apical interface where PATJ is enriched. We denote the binding strength at this interface by ϵ_{int} and binding strength to the membrane far from this interface by ϵ_0. The interface width is denoted χ. To initiate condensates, we introduce nucleation sites at positions $\vec{x}_i$, where $i = 1, \ldots, N$, where N is the number of sites. The binding affinity to nucleation sites is ϵ_{nuc} and their width is denoted χ_i. We therefore write

$$\epsilon(\vec{x}) = \epsilon_0 + (\epsilon_{int} - \epsilon_0) \exp\left(-\frac{(z-z_{int})^2}{2\chi^2}\right) + \sum_{i=1}^{N} \epsilon_{nuc} \exp\left(-\frac{(\vec{x}-\vec{x_i})^2}{2\chi_i^2}\right) \quad , \quad (3)$$

Here z is the position coordinate along the apical-lateral axis and z_{int} denotes the position of the apical interface on the membrane.

The chemical potential of proteins on the membrane, $\mu = \sigma \delta F / \delta\phi$, reads

$$\mu = -r\phi + \lambda\phi^3 - \epsilon - \kappa\nabla^2\phi \quad . \quad (4)$$

For simplicity, we use the same parameter values and binding affinity at the apical and lateral domains. Below we discuss the dynamics of nucleation and elongation of ZO-1 protein condensates described by this model.

Elongation dynamics of ZO-1 condensates

In order to describe the protein dynamics on the membrane driven by recruitment of proteins from the bulk, we consider the Allen-Cahn equation[8] for the composition variable ϕ:

$$\frac{\partial\phi}{\partial t} = \gamma(\mu_{bulk} - \mu) \quad , \quad (5)$$

where γ is a kinetic coefficient. The composition variable increases when proteins are recruited from solution and $\mu_{bulk} > \mu$. It decreases when $\mu_{bulk} < \mu$. At steady state $\mu = \mu_{bulk}$. The steady state condition is satisfied both for the condensed phase with $\phi = \phi_c$, as well as for the dilute phase with $\phi = \phi_d$. At the edge of a growing condensate where material is recruited, we have $\mu_{bulk} > \mu$ (Extended Data Fig.5 d, e, j, k).

We solve Eq. 5 numerically and find that once condensates are nucleated at the predetermined nucleation sites $\vec{x_i}$, they subsequently elongate along the apical interface at constant velocity v if the binding affinity ϵ_{int} is sufficiently strong. Varying this binding affinity, we find that the elongation velocity v increases for increasing binding affinity (Fig. 4g, h). Furthermore, condensate elongation only happens for ϵ_{int} larger than a threshold value ϵ_t , or in terms of the relative binding affinity, $\Delta\epsilon = \epsilon + \mu_{bulk}$, for $\Delta\epsilon > \Delta\epsilon_t$. Below this threshold, condensates nucleate and grow to a limited size but do not elongate along the apical interface (Extended Data Fig. 5 e, d (lower panel).

When multiple condensates are nucleated at the same time on the apical interface, the process to form a condensate covering the whole apical interface is faster, while the elongation speed of individual condensates is unchanged. In such a process the elongation of condensates jumps discontinuously when condensates fuse (Extended Data Fig. 5g-i). In all numerical calculations we vary the binding affinity to the interface, ϵ_{int}, and keep the other parameters fixed (see Extended Data figure 5 l)

Elongation speed in a one-dimensional system

We can calculate the elongation speed of condensates in an infinite one-dimensional system[9,10] To this end, we write the dynamic equation for the composition variable along the centerline $z = z_{int}$ of the apical interface, where $\epsilon = \epsilon_{int}$:

$$\frac{\partial\phi}{\partial t} = -\gamma\left(\lambda\phi^3 - r\phi + \Delta\epsilon - \kappa\frac{\partial^2\phi}{\partial x^2}\right) \quad , \quad (6)$$

here we defined $\Delta\epsilon = \epsilon_{int} + \mu_{bulk}$. An elongating condensate can be described by a front profile that connects the condensed to the dilute phase. We seek solutions of fronts $\phi = \phi(x - vt)$ moving at constant velocity v. In

order to determine this profile as well as the velocity v, we express Eq. 6 in the reference frame that is co-moving with the front. In this reference frame, the variable $\xi = x - vt$ measures the distance to the front. Eq. 6 then becomes

$$-v\frac{\partial\phi}{\partial\xi} + \gamma\left(\lambda\phi^3 - r\phi + \Delta\epsilon - \kappa\frac{\partial^2\phi}{\partial x^2}\right) \quad . \qquad (7)$$

In order to solve this equation, we write the cubic polynomial in terms of its roots ϕ_c, ϕ_d and ϕ^*. We then have

$$\lambda\phi^3 - r\phi + \Delta\epsilon = \lambda(\phi - \phi_c)(\phi - \phi_d)(\phi - \phi^*) \quad . \qquad (8)$$

The roots can be expressed explicitly as

$$\phi_c = 2\sqrt{\frac{r}{3\lambda}}\cos\left[\frac{1}{3}\cos^{-1}\left(\frac{3\Delta\epsilon}{2r}\sqrt{\frac{3\lambda}{r}}\right)\right] \quad ,$$

$$\phi_d = 2\sqrt{\frac{r}{3\lambda}}\cos\left[\frac{1}{3}\cos^{-1}\left(\frac{3\Delta\epsilon}{2r}\sqrt{\frac{3\lambda}{r}}\right) - \frac{4\pi}{3}\right] \quad ,$$

$$\phi^* = 2\sqrt{\frac{r}{3\lambda}}\cos\left[\frac{1}{3}\cos^{-1}\left(\frac{3\Delta\epsilon}{2r}\sqrt{\frac{3\lambda}{r}}\right) - \frac{2\pi}{3}\right] \quad . \qquad (9)$$

These roots are stationary values of the composition ϕ of the dynamic equation, Eq. 6. The values ϕ_c and ϕ_d are the stable steady state solutions corresponding to a condensed phase and to a dilute phase, respectively. The value ϕ^* is an unstable steady state.

In order to determine the velocity v, we use the ansatz

$$\frac{\partial\phi}{\partial\xi} = A(\phi_c - \phi)(\phi - \phi_d) \quad , \qquad (10)$$

where A is a constant to be determined. Here $\phi(\xi)$ has a vanishing derivative for $\phi = \phi_d$ or $\phi = \phi_c$, representing a front profile that connects a condensed and a dilute phase. Using this ansatz in Eq. 7, leads to

$$(\phi_c - \phi)(\phi - \phi_d)(Av + A^2\gamma\kappa(\phi_c + \phi_d) - \gamma\lambda\phi^* + \gamma(-2A^2\kappa + \lambda)\phi) = 0 \quad . \qquad (11)$$

This relation needs to be satisfied for any value of ϕ, which requires

$$A = -\sqrt{\frac{\lambda}{2\kappa}} \quad . \qquad (12)$$

From this follows the value of the velocity

$$v = \gamma\sqrt{\frac{\kappa\lambda}{2}}(\phi_c + \phi_d - 2\phi^*) \quad . \qquad (13)$$

Integrating Eq. 10, we obtain the front profile

$$\phi(\xi) = \frac{\phi_c+\phi_d}{2} + \frac{\phi_d-\phi_c}{2}\tanh\left[\frac{\sqrt{\lambda}(\phi_c-\phi_d)\xi}{2\sqrt{2}\kappa}\right] \quad . \qquad (14)$$

This profile transitions from the condensed phase for $\xi < 0$ to the dilute phase $\xi > 0$ over the width of the front, $l = \sqrt{\lambda}(\phi_c - \phi_d)/(2\sqrt{2}\kappa)$.

Using the expressions Eq. (9) and (13) we show that the elongation velocity v as a function of relative binding affinity $\Delta\epsilon$ is an increasing function (Extended Data Fig. 5e,f). In this case the velocity can be both negative and positive, depending on the sign of $\Delta\epsilon$, corresponding to elongating and shrinking condensates, respectively. This can be compared to the elongation velocity in the case of pre-nucleated condensates in 2D, which is always positive but requires a binding affinity relative to the chemical potential of the bulk above a threshold value (Extended Data Fig.5f).

Supplementary Note 2. Time-resolved proximity proteomics of junctional condensates

Time resolved APEX2 has been instrumental in systematically dissecting the molecular composition of condensates due to its fast kinetics[11,12]. Combining APEX2 protein profiling with a calcium switch assay we profiled the ZO-1 proteome over the tight junction assembly. Control experiments confirmed correct tight junction

localization of the ZO1-APEX2 fusion construct and the biotinylating activity of APEX2 at the tight junction (Fig. 1a, Extended data Fig. 1a-d).

Fluorescence imaging of stably expressing Dendra2-ZO1-APEX2 in MDCK-II kidney epithelia monolayers confirmed correct tight junction localization of the fusion construct (Extended data Fig. 1b-c). Next, we confirmed the proximity labelling activity of the APEX2 enzyme by incubating the tissue with biotin-phenol for 30 min and subsequently induced the proximity labelling reaction by a short 1 min pulse of hydrogen peroxide (Extended Data Fig.1a). After quenching, fixation and staining with fluorescent streptavidin, two color imaging showed that the biotinylated proteins were highly enriched at the tight junction zone (Extended Data Fig.1c), demonstrating that Dendra2-ZO1-APEX2 proximity biotinylating provides high spatiotemporal contrast for mapping tight junction assembly. We further confirmed the APEX2 activity by blotting the biotinylated pull-down proteins (Extended Data Fig.1d).

Synchronized tight junction assembly was induced by sudden restoration of physiological calcium levels after overnight calcium depletion (Fig. 1a). In line with previous studies, we found that formation of a closed ZO-1 belt surrounding each cell took around 3h to 5h (Fig. 1a, Supplementary Video 1)[4,13]. We performed ZO-1 proteomics proximity-labelling at 0h, 0.5h, 1h, 3h and 18h after calcium switch, by activating APEX2 with a 1 min pulses of hydrogen peroxide, followed by isolation of the proteins via streptavidin pull down and digestion into tryptic peptides (Extended Data Fig.1f). To process all time-points in the same quantitative mass-spec analysis, we used a multiplex proteomic approach based on tandem mass tag (TMT). The TMT isobaric tagging approach enables robust quantitative proteomics by measuring all samples in one run. Hence, statistical analysis of relative protein enrichment at different time- points after calcium switch was possible across proteins detected in all time-points (Extended Data Fig.1g-i). To analyze the changes of the ZO-1 interactome as a function of tight junction assembly time, we calculated the fold change (FC) of protein abundance with respect to the time point zero, i.e. the calcium depleted state with dissociated junctions and cytoplasmic localization of ZO-1. Proteins with FC > 2 were considered as potential hits and a false discovery rate threshold of 0.05 was used to filter out noisy data. In addition, we excluded false positive hits due to non- junctional interactions (ribosomes, nucleus, mitochondria, ER, see Supplementary Table 3). The results of the analysis are shown as volcano plots in (Fig. 1b). Proteins that significantly increased (hits) with respect to the dissociated state are highlighted in red and selected tight junction proteins are annotated. The protein proximity analysis revealed that the junctional condensates increase in molecular complexity during the assembly process (Fig.1b). We found that the majority of the proteins appeared after ZO-1 membrane condensates formed around 0.5h, showing that initial composition of ZO-1 condensates is strongly remodeled over time. The most significant increase in junctional components occurred after 1h, which corresponds to the time when the tight junction belt starts to close in live cell imaging (Fig. 1a).

Supplementary Note 3. Recruitment kinetics reveal polarization of junctional condensates

Earlier work on junction assembly suggested that the nucleation stage of tight junctions involves the recruitment of ZO proteins to adherens junctions via alpha-catenin[14,15]. Interestingly, our time-resolved ZO-1 interactome does not support this view. Our data rather suggest that interactions with cadherin complexes (CDH6) are

established at a later stage possibly via afadin (3-18h). In addition, we found that the RNA-binding protein YBX3 (ZONAB), which regulates cell proliferation, is sequestered to ZO-1 condensates very early (0.5h) but is gradually excluded from ZO-1 interactions as the junction matures, indicating a rather transient role in signaling[16,17]. At the final time point (18h) the majority of interaction partners around ZO-1 show a stable enrichment with a few exceptions, which abundance decreased again at the last stage (SHROOMs, CLDN4, NECTIN2) (Fig.1 d,e).

Supplementary Note 4. Imaging junctional recruitment dynamics

We chose one candidate from the early (ZO-2), intermediate (MAGI-3) and late (PATJ) stage and used CRISPR/Cas9 to create fluorescent reporters with mScarlet (mS), respectively (Extended Data Fig. 2a). Fluorescent tagging of candidate proteins was done in a MDCK-II cell line which expressed mNeoGreen (mN) tagged endogenous ZO-1, to enable 2 color imaging of junction assembly. Sequencing confirmed homozygous insertion of the tags (Extended Data Fig. 2a) and imaging confirmed that mS tagging of the endogenous proteins resulted in proper co-localization with mN-ZO-1 at the tight junction belt in confluent monolayers (Extended Data Fig. 2a).

Next, we performed live-imaging of the 2-color cell lines using the calcium switch assay to quantify protein arrival kinetics during tight junction assembly for 3 hours (Extended Data Fig. 2b). To determine the arrival kinetics of the mS-tagged tight junction proteins we segmented the mN-ZO-1 signal and quantified the mS intensity in the segmented ZO-1 condensates and the cytoplasm over time (Extended Data Fig. 2d). To directly correct for photo-bleaching artefacts, we calculate the ratio between the junctional and the cytoplasmic mS signal for each time point (Extended Data Fig.2d). Assuming that bleaching is spatially homogenous the ratio is independent of bleaching and directly reports the enrichment of the protein in the condensed ZO-1 phase (tight junction). The kinetics of the junction enrichment ratio showed that mS-ZO2 was rapidly enriched after ZO-1 condensation (Extended Data Fig. 2c). In comparison, mS-MAGI3 was recruited slower and reached saturation later. Finally, mS-PATJ recruitment was delayed even longer and showed a visible enrichment only around the time of junction spreading (Extended Data Fig. 2b, c). In order to calculate the half times ($t_{1/2}$) of client protein arrival we fitted the kinetic data to a HillSlope model (Extended Data Fig.3c) and determined the difference in arrival time between ZO-1 and the client protein in living cells. The analysis confirmed the trend of the recruitment kinetics observed in the proximity proteomics dataset with early ZO2 (~5 min), intermediate MAGI3 (~18 min) and late PATJ (~35 min) (Fig. 2c).

Supplementary References

1. Safran, S. A. *Statistical Thermodynamics of Surfaces, Interfaces, and Membranes*. (CRC Press, 2018). doi:10.1201/9780429497131.

2. de Gennes, Françoise Brochard-Wyart & David Quéré. Capillarity and Wetting Phenomena: Drops, Bubbles, Pearls, Waves. *Phys Today* **57**, 66–67 (2004).

3. Berthier, J., Gosselin, D. & Berthier, E. A generalization of the Lucas–Washburn–Rideal law to composite microchannels of arbitrary cross section. *Microfluid Nanofluidics* **19**, 497–507 (2015).

4. Beutel, O., Maraspini, R., Pombo-García, K., Martin-Lemaitre, C. & Honigmann, A. Phase Separation of Zonula Occludens Proteins Drives Formation of Tight Junctions. *Cell* **179**, 923-936.e11 (2019).

5. Rouches, M., Veatch, S. L. & Machta, B. B. Surface densities prewet a near-critical membrane. *Proceedings of the National Academy of Sciences* **118**, e2103401118 (2021).

6. Rouaud, F. *et al.* Cingulin and paracingulin tether myosins-2 to junctions to mechanoregulate the plasma membrane. *Journal of Cell Biology* **222**, e202208065 (2023).

7. Shen, L., Weber, C. R. & Turner, J. R. The tight junction protein complex undergoes rapid and continuous molecular remodeling at steady state. *Journal of Cell Biology* **181**, 683–695 (2008).

8. Allen, S. M. & Cahn, J. W. A microscopic theory for antiphase boundary motion and its application to antiphase domain coarsening. *Acta Metallurgica* **27**, 1085–1095 (1979).

9. Ben-Jacob, E., Brand, H., Dee, G., Kramer, L. & Langer, J. S. Pattern propagation in nonlinear dissipative systems. *Physica D* **14**, 348–364 (1985).

10. Stegemerten, F., Gurevich, S. V & Thiele, U. Bifurcations of front motion in passive and active Allen–Cahn-type equations. *Chaos: An Interdisciplinary Journal of Nonlinear Science* **30**, 053136 (2020).

11. Padrón, A., Iwasaki, S. & Ingolia, N. T. Proximity RNA Labeling by APEX-Seq Reveals the Organization of Translation Initiation Complexes and Repressive RNA Granules. *Mol Cell* **75**, 875-887.e5 (2019).

12. Markmiller, S. *et al.* Context-Dependent and Disease-Specific Diversity in Protein Interactions within Stress Granules. *Cell* **172**, 590-604.e13 (2018).

13. Riesen, F. K., Rothen-Rutishauser, B. & Wunderli-Allenspach, H. A ZO1-GFP fusion protein to study the dynamics of tight junctions in living cells. *Histochem Cell Biol* **117**, 307–315 (2002).

14. Hartsock, A. & Nelson, W. J. Adherens and tight junctions: Structure, function and connections to the actin cytoskeleton. *Biochim Biophys Acta Biomembr* **1778**, 660–669 (2008).

15. Rajasekaran, A. K., Hojo, M., Huima, T. & Rodriguez-Boulan, E. Catenins and zonula occludens-1 form a complex during early stages in the assembly of tight junctions. *Journal of Cell Biology* **132**, 451–463 (1996).

16. Spadaro, D. *et al.* ZO proteins redundantly regulate the transcription factor DbpA/ZONAB. *Journal of Biological Chemistry* **289**, 22500–22511 (2014).

17. Balda, M. S., Garrett, M. D. & Matter, K. The ZO-1-associated Y-box factor ZONAB regulates epithelial cell proliferation and cell density. *Journal of Cell Biology* **160**, 423–432 (2003).
