# Supplementary material for: Membrane prewetting by condensates promotes tight-junction belt formation
Source: Nature. 2024 Aug 7;632(8025):647–55. doi: 10.1038/s41586-024-07726-0 (PMC11324514; doi:10.1038/s41586-024-07726-0)

nature portfolio

Corresponding author(s): Karina Pombo-Garcia
Alf honigmann

Last updated by author(s): 2024-05-10

Reporting Summary

Nature Portfolio wishes to improve the reproducibility of the work that we publish. This form provides structure for consistency and transparency in reporting. For further information on Nature Portfolio policies, see our Editorial Policies and the Editorial Policy Checklist.

Statistics

For all statistical analyses, confirm that the following items are present in the figure legend, table legend, main text, or Methods section.

n/a	Confirmed	
☐	☒	The exact sample size (*n*) for each experimental group/condition, given as a discrete number and unit of measurement
☐	☒	A statement on whether measurements were taken from distinct samples or whether the same sample was measured repeatedly
☐	☒	The statistical test(s) used AND whether they are one- or two-sided<br>*Only common tests should be described solely by name; describe more complex techniques in the Methods section.*
☒	☐	A description of all covariates tested
☐	☒	A description of any assumptions or corrections, such as tests of normality and adjustment for multiple comparisons
☐	☒	A full description of the statistical parameters including central tendency (e.g. means) or other basic estimates (e.g. regression coefficient) AND variation (e.g. standard deviation) or associated estimates of uncertainty (e.g. confidence intervals)
☐	☒	For null hypothesis testing, the test statistic (e.g. *F*, *t*, *r*) with confidence intervals, effect sizes, degrees of freedom and *P* value noted<br>*Give P values as exact values whenever suitable.*
☒	☐	For Bayesian analysis, information on the choice of priors and Markov chain Monte Carlo settings
☒	☐	For hierarchical and complex designs, identification of the appropriate level for tests and full reporting of outcomes
☒	☐	Estimates of effect sizes (e.g. Cohen's *d*, Pearson's *r*), indicating how they were calculated

Our web collection on statistics for biologists contains articles on many of the points above.

Software and code

Policy information about availability of computer code

Data collection

STED, confocal and FRAP imaging on Abberior operated by software Imspector (16.2.8415), Live imaging on DeltaVision Elite operated by Resolve3D softWoRx-Acquire (Ver 7.2.0). Mass spectrometry (MS) data was collected using an Orbitrap Fusion'" Lumos'" Tribrid'" Mass Spectrometer (Thermo Fischer Scientific). Proteomics raw data files were processed using IsobarQuant Package on Python https://github.com/protcode/isob and Mascot (ver. 2.4, MatrixScience). Data were searched against Canis Lupus Uniprot database (UP000805418), Wblot fluorescence on Image studio software (ver 5.2.5).

Data analysis

Image analysis: Fiji(ImageJ 1.53) or segmentation imaging analysis done with Custom scripts in Matlab (ver. R2019a), Plot and statistics Graphpad Prism (9.4.1). CellPose was used for segmentation (https://github.com/mouseland/cellpose) and ZO-1 condensates segmentation using the plugin Skeleton in Fiji. Extension analysis was done using "JFilament" in Fiji and further quantify using "JFilament" tack on Matlab. Sample registration was done using StackReg in Fiji. Eccentricity was calculated by Matlab function "regionprops "

Proteomics analysis : R programming language (ISBN 3-900051-07-0) ,Rstudio(ver 1.1.419),vsnfor R (ver 3.9), limma for R (ver 3.9), fdrtool for R (ver. 1.2.15), ggplot2 R package was used to generate the graphical, based on previous available code at https/ /github.com/fstein/EcoliTPP .
packages:
limma (Ritchie et al., 2015), https://bioconductor.org/packages/limma/
vsn (Huber et al., 2002), https://bioconductor.org/packages/vsn/
MSnbase (Gatto et al., 2012), https://bioconductor.org/packages/MSnbase/
tidyverse (Wickham et al., 2019), https://tidyverse.tidyverse.org/
biobroom (Bass et al., 2015), https://bioconductor.org/packages/biobroom/
ass et al., 2015), https://bioconductor.org/packages/biobroom/
[illegible]

ggrepel (Slowikowski et al., 2018), https://cran.r-project.org/web/packages/ggrepel/vignettes/ggrepel.html
ClusterProfiler (Yu, et al., 2012), https://bioconductor.org/packages/clusterProfiler/
Interactome created on Cytoscape (ver 3.9.0) using a STRING database (ver 11.5). Cell localization gen ontology annotation from http://geneontology.org

Numerical calculations were done using programming language: Python 3.8.10, all codes were run using IPython 7.3.10. This software comes pre-installed in most of the Linux distributions. OS Name: Ubuntu 20.04.6 LTS, OS Type: 64-bit, GNOME Version: 3.36.8. All codes and a minimal dataset can be found in Github (https://doi.org/10.5281/zenodo.11174401).

For manuscripts utilizing custom algorithms or software that are central to the research but not yet described in published literature, software must be made available to editors and reviewers. We strongly encourage code deposition in a community repository (e.g. GitHub). See the Nature Portfolio guidelines for submitting code & software for further information.

Data

Policy information about availability of data

All manuscripts must include a data availability statement. This statement should provide the following information, where applicable:

- Accession codes, unique identifiers, or web links for publicly available datasets
- A description of any restrictions on data availability
- For clinical datasets or third party data, please ensure that the statement adheres to our policy

All the data supporting the findings of this study is available within the article in the source data, figures and extended data, core code produce for the analysis including STED analysis, recruitment kinetics and numerical calculations for the thermodynamic model is available with a minimal dataset on Github ascession code https://doi.org/10.5281/zenodo.11174401, MS data was analyzed using uniprot canis lupus proteome database (UP000805418). Data are available via ProteomeXchange with identifier PXD052221

Research involving human participants, their data, or biological material

Policy information about studies with human participants or human data. See also policy information about sex, gender (identity/presentation), and sexual orientation and race, ethnicity and racism.

Reporting on sex and gender	N/A
Reporting on race, ethnicity, or other socially relevant groupings	N/A
Population characteristics	N/A
Recruitment	N/A
Ethics oversight	N/A

Note that full information on the approval of the study protocol must also be provided in the manuscript.

Field-specific reporting

Please select the one below that is the best fit for your research. If you are not sure, read the appropriate sections before making your selection.

☒ Life sciences ☐ Behavioural & social sciences ☐ Ecological, evolutionary & environmental sciences

For a reference copy of the document with all sections, see nature.com/documents/nr-reporting-summary-flat.pdf

Life sciences study design

All studies must disclose on these points even when the disclosure is negative.

Sample size	"No statistical method was applied to predetermine sample size. Proteomics experiments sampled over 1000 cells per time point, which was sufficient to reproduce protein enrichment values between experimental repeats. Tissue culture imaging experiments were performed on large field of views to sample over 40 cells per experiment, which was sufficient to obtain reproducible mean values between experimental repeats. "
Data exclusions	We excluded false positive data from the APEX2 volcano plot for visualizations purposes. False positives were identified based on previous annotation of the respective protein to other organelles (mitochondria, ER, ribosomes, etc).
Replication	All imaging experiments were replicated at least 3 times. The exact number of independent replicates is given in the figure. For all experiments replication attempts were successful.
Randomization	Randomization was not necessary for mass spectrometry (MS) experiment, as the library preparation was done by multiplexing different samples using TMT isobaric labeling and therefore all samples were simultaneously analyzed in the same MS run for each biological replicate. For the imaging analysis areas of the monolayer were randomly imaged . For the extension analysis the cell handling and imaging was done by different researchers.
Blinding	No blinding was performed for imaging experiments. Proteomics, bio-informatics and qPCR were blinded in the sense that the scientists at the facilities that performed the experiments were not familiar the meaning of the sample names.

Reporting for specific materials, systems and methods

We require information from authors about some types of materials, experimental systems and methods used in many studies. Here, indicate whether each material, system or method listed is relevant to your study. If you are not sure if a list item applies to your research, read the appropriate section before selecting a response.

Materials & experimental systems

n/a	Involved in the study
☐	☒ Antibodies
☐	☒ Eukaryotic cell lines
☒	☐ Palaeontology and archaeology
☒	☐ Animals and other organisms
☒	☐ Clinical data
☒	☐ Dual use research of concern
☒	☐ Plants

Methods

n/a	Involved in the study
☒	☐ ChIP-seq
☒	☐ Flow cytometry
☒	☐ MRI-based neuroimaging

Antibodies

Antibodies used

Primary
1. ZO-1 mouse monoclonal (Invitrogen, 33-9100, clon 1A12), dilution WB (1:200)
2. PATJ (Nterm) rabbit polyclonal (gifted house produced), dilution WB (1:200)
3. PATJ (PDZ4) rabbit polyclonal (LSbio, LC-C410011), dilution WB (1:200), IF (1:50)
4. PALS1 mouse monoclonal (santa cruz, sc-365411, clon G5), dilution IF (1:50)
5. Occludin rabbit polyclonal (life tec, 71-1500), dilution IF (1:50)
6. Lin7 rabbit polyclonal (thermofisher, 51-5600), dilution IF (1:50)
7. E-Cadherin rabbit monoclonal (Cell signalling, 3195S, clon 24E10), dilution IF (1:50)
8. Beta-actin rabbit polyclonal (abacam, ab8227), dilution WB (1:500)

Secondary
1. goat anti-mouse star-red (Abberior, star-red 2-0002-011-2), dilution IF (1:200)
2. goat anti-rabbit star-orange (Abberior, storange-1102), dilution IF (1:200)
3. goat anti rabbit IgG-HRP (H+L) (Cell Signaling, #7074), dilution WB (1:5000)
4. goat anti-Mouse IgG-HRP (H+L) (Cell Signaling, #7076), dilution WB (1:5000)
5. IRDye-800CW Streptavidin (LI-COR, 92632230), dilution IF (1:5000)

Validation

Validation
We have used antibodies that have been validated by the commercial provider and several publications from scientific labs working on tight junctions. We provide as example of each a key literature reference and the validation by the provider.
1. ZO-1 mouse monoclonal (Invitrogen, 33-9100, clon 1A12), dilution WB (1:200)
Ref paper: doi: 10.1083/jcb.202001042
Supplier: The specificity of the antibody was validated by the supplier checking in knock down of endogenous ZO-1 in Caco-2 cells and staining on MDCK-II.
2. PATJ (Nterm) rabbit polyclonal (gifted house produced), dilution WB (1:200)
PATJ L27 Nterm rabbit polyclonal gift from Le Bivic lab (DOI10.1074/jbc.M202196200). This antibody was generated on the paper were they originally renamed hINADl to PATJ as protein associated to tight junctions. They confirm the antibody in Caco-2 and in MDCK-II (same cells used in this study) reporting by western blot a size ~ 230KDa and an apical localization to tight junctions. They also confirmed it on knock down cells (doi:10.1242/jcs.02528)
3. PATJ (PDZ4) rabbit polyclonal (LSbio, LC-C410011), dilution WB (1:200), IF (1:50)
Ref. paper: https://doi.org/10.1016/j.cub.2020.05.032 (IF and WB on MDCK-II)
Supplier: The specificity of the antibody was validated by the supplier via WB on A431 cells
4. PALS1 (Nterm) mouse monoclonal (santa cruz, sc-365411, clon G5), dilution IF (1:50)
Ref. paper: doi: 10.1016/j.jcmgh.2021.01.022. and https://doi.org/10.1016/j.cub.2020.05.032 (IF and WB on MDCK-II)
Supplier: The specificity of the antibody was validated by the supplier via WB on A431 cells
5. Occludin rabbit polyclonal (life tec, 71-1500), dilution IF (1:50)
Ref. paper: 10.1091/mbc.E11-08-0681
Supplier: The specificity of the antibody was validated by the supplier via WB on MDCK-II cells via IF and WB and also on cells with the endogenous gen ko
6. Lin7 rabbit polyclonal (thermofisher, 51-5600), dilution IF (1:50)
Ref. paper: https://doi.org/10.1016/j.cub.2020.05.032 (IF and WB on MDCK-II) and 10.1091/mbc.e09-04-0280
Supplier: The specificity of the antibody was validated by the supplier via WB MDCK-II cells
7. E-Cadherin rabbit monoclonal (Cell signalling, 3195S, clon 24E10), dilution IF (1:50)
Ref. paper: 10.1038/s41467-017-01145-8
Supplier: The specificity of the antibody was validated by the supplier checking the lack of fluorescence in the absence of mouse IgG antibodies.
8. Beta-actin rabbit polyclonal (abacam, ab8227), dilution WB (1:500)
Ref. paper: 10.1091/mbc.E20-05-0291
Supplier: The specificity of the antibody was validated by the supplier in MDCK-II via WB

Eukaryotic cell lines

Policy information about cell lines and Sex and Gender in Research

Cell line source(s)	MDCK-II were purchased from (00062107, Public Health England) , HEK293 (85120602,Public Health England)
Authentication	Certificate of analysis of these cell lines were provided by the vendors.<br>For generation of genetically modify knock in or knock out, different levels of identification were done: genotyping via PCR, sequencing on extracted genomic DNA, inmunostaining and inmunoblotting, and the edited cells were indicated by a distinct PCR band, correct sequencing results or a inmunofluorescence signal. Some cell lines were used by other lab members and reported before (https://doi.org/10.1016/j.cell.2019.10.011)
Mycoplasma contamination	All cell lines used were checked against Mycoplasma contamination every 3 months using universal mycoplasma kit detection and send for sequence and confirmed negative.
Commonly misidentified lines<br>(See ICLAC register)	no commonly misidentified lines were used

Plants

Seed stocks	*Report on the source of all seed stocks or other plant material used. If applicable, state the seed stock centre and catalogue number. If plant specimens were collected from the field, describe the collection location, date and sampling procedures.*
Novel plant genotypes	*Describe the methods by which all novel plant genotypes were produced. This includes those generated by transgenic approaches, gene editing, chemical/radiation-based mutagenesis and hybridization. For transgenic lines, describe the transformation method, the number of independent lines analyzed and the generation upon which experiments were performed. For gene-edited lines, describe the editor used, the endogenous sequence targeted for editing, the targeting guide RNA sequence (if applicable) and how the editor was applied.*
Authentication	*Describe any authentication procedures for each seed stock used or novel genotype generated. Describe any experiments used to assess the effect of a mutation and, where applicable, how potential secondary effects (e.g. second site T-DNA insertions, mosiacism, off-target gene editing) were examined.*

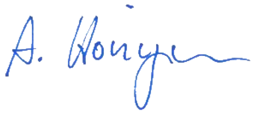

Supplement: Supplementary file 2 — Reporting Summary [file 41586_2024_7726_MOESM2_ESM.pdf]
