# Supplementary material for: Membrane prewetting by condensates promotes tight-junction belt formation
Source: Nature. 2024 Aug 7;632(8025):647–55. doi: 10.1038/s41586-024-07726-0 (PMC11324514; doi:10.1038/s41586-024-07726-0)
Supplement: Supplementary file 3 — Uncropped western blots. [file 41586_2024_7726_MOESM3_ESM.pdf]

Supplementary Figure 1

Uncropped blots

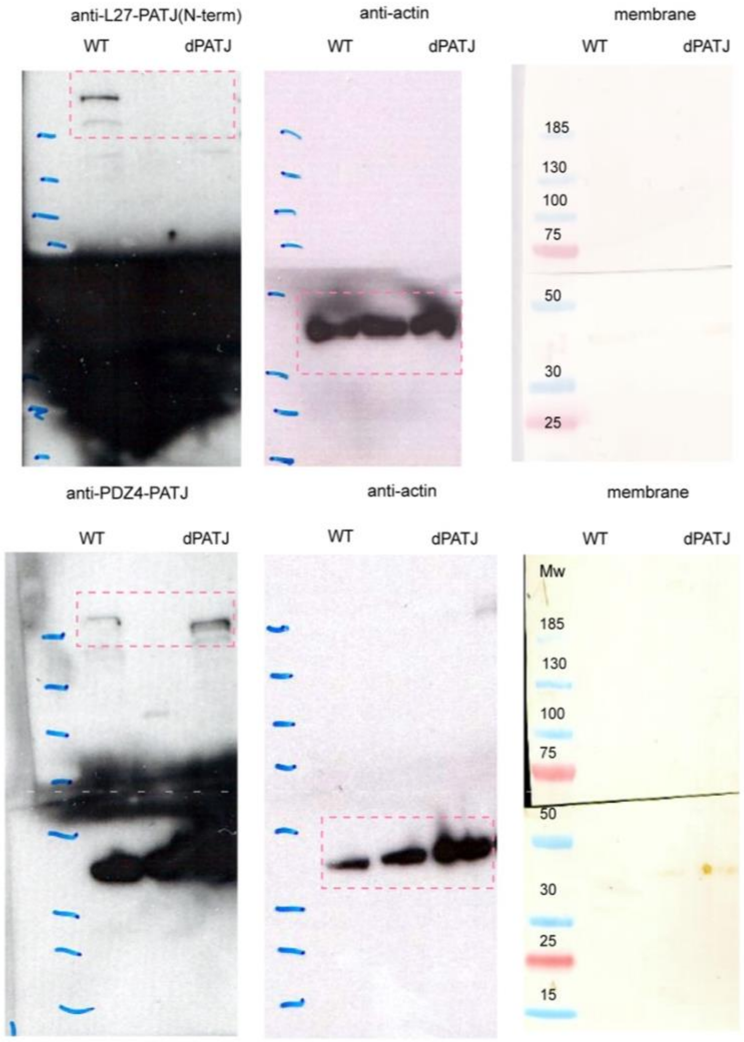

Supplementary Figure 1 shows the uncropped blots performed to verify the levels of PATJ on the WT and dL27-PATJ MDCK-II. Western blots were performed using classical HRP method and the shown images are the original full films. The upper panel shows the WB against L27 domain, the deleted one, not present in the dL27-PATJ MDCK-II cells. From left to right the blot shows the L27 blot expose for 2 minutes, the middle shows the actin control, run on the same gel same membrane but different film exposed 30 seconds, last image shows the membrane with the markers. The lower bottom panel shows the blot against PATJ-PDZ4 with a ~40kDa shifted in size on the dL27-PATJ MDCK-II cells, middle is the actin control and right the membrane. Both membranes in the middle were cut to avoid antibody cross reactivity (See supplementary methods and Extended Data figure 4b.
